# Comparative parallel multi-omics analysis during the induction of pluripotent and trophectoderm states

**DOI:** 10.1038/s41467-022-31131-8

**Published:** 2022-06-17

**Authors:** Mohammad Jaber, Ahmed Radwan, Netanel Loyfer, Mufeed Abdeen, Shulamit Sebban, Areej Khatib, Hazar Yassen, Thorsten Kolb, Marc Zapatka, Kirill Makedonski, Aurelie Ernst, Tommy Kaplan, Yosef Buganim

**Affiliations:** 1 Department of Developmental Biology and Cancer Research, Institute for Medical Research Israel-Canada, The Hebrew University-Hadassah Medical School, Jerusalem, 91120 Israel; 2 School of Computer Science and Engineering, The Hebrew University of Jerusalem, Jerusalem, 9190401 Israel; 3 Group Genome Instability in Tumors, DKFZ, Heidelberg, Germany; 4 German Cancer Consortium (DKTK), 69120 Heidelberg, Germany; 5 Division of Molecular Genetics, German Cancer Research Consortium (DKTK), German Cancer Research Center (DKFZ), 69120 Heidelberg, Germany

**Keywords:** Reprogramming, Multipotent stem cells, Stem cells

## Abstract

Following fertilization, it is only at the 32-64-cell stage when a clear segregation between cells of the inner cell mass and trophectoderm is observed, suggesting a ‘T’-shaped model of specification. Here, we examine whether the acquisition of these two states in vitro, by nuclear reprogramming, share similar dynamics/trajectories. Using a comparative parallel multi-omics analysis (i.e., bulk RNA-seq, scRNA-seq, ATAC-seq, ChIP-seq, RRBS and CNVs) on cells undergoing reprogramming to pluripotency and TSC state we show that each reprogramming system exhibits specific trajectories from the onset of the process, suggesting ‘V’-shaped model. We describe in detail the various trajectories toward the two states and illuminate reprogramming stage-specific markers, blockers, facilitators and TSC subpopulations. Finally, we show that while the acquisition of the TSC state involves the silencing of embryonic programs by DNA methylation, during the acquisition of pluripotency these regions are initially defined but retain inactive by the elimination of H3K27ac.

## Abstract (web summary)

Ectopic transcription factor expression can reprogram mouse fibroblasts to pluripotent or trophoblast stem cells. Here the authors apply multi-omics analyses to the induction of pluripotency and trophoblast stem cell state from fibroblasts, comparing these with the changes in transcriptome of early embryonic cells.

## Introduction

Fertilization of an oocyte initiates robust epigenetic reprogramming of the DNA content within the newly formed cell, resulting in a totipotent zygote having the potential to produce all embryonic and extra-embryonic tissues^1^. Several divisions later, an early blastocyst is formed, containing two more committed compartments: an inner cell mass (ICM) which contains pluripotent cells (epiblast (Epi)) that will form the embryo proper, and an outer layer of trophectoderm (TE) cells, which will become components of extra-embryonic tissues such as the placenta^2–4^.

Exactly how the specification into ICM and TE cells occurs is not fully understood, although several models have been suggested^2–4^. Recently, the transcriptional trajectory from zygote to blastocyst has been described using single-cell transcriptomic data for both human and mouse^5–11^. Interestingly, while there are notable differences between humans and mice, key developmental aspects in the first cell fate decision process are shared by both. In both, clear transcriptional changes are found between stages (i.e., zygote, 2-cell stage, 4-cell stage, 8-cell stage, morula, and blastocyst) but with minimal transcriptional heterogeneity within cells of each stage before blastocyst formation. Although some genes, like *Sox21*, were shown to exhibit transcriptional heterogeneity even within the 4-cell mouse embryo^6^, the overall transcriptome is relatively similar between the four cells. This suggests a ‘T’-shaped model, where cells undergo similar transcriptional changes before segregation, and separate into two distinct cell types, ICM and TE, only at the morula/early blastocyst stage. This notion is supported by the ability of 2–8-cell stage blastomeres to become both TE and ICM, and by the observation that cells of the outer layer of the morula can migrate into the inner layer and become pluripotent cells, suggesting dynamic chromatin landscape and transcriptome, that are relatively analogous between the cells before the final specification^3,4^.

Epigenetic reprogramming of a somatic nucleus to pluripotency or to a TE state has been achieved in vitro by somatic cell nuclear transfer (SCNT^12^) or by forced expression of a defined number of transcription factors^13–19^. While ectopic expression of *Oct4*, *Sox2*, *Klf4*, and *Myc* (OSKM) induces the formation of pluripotent stem cells (PSC, the in vitro counterpart of the ICM-Epi^17^), we and others have shown that ectopic expression of *Gata3*, *Eomes*, *Tfap2c* and *Myc* (GETM, or *Ets2* instead of *Myc*) induces the formation of trophoblast stem cells (TSC, the in vitro counterpart of the TE) from mouse fibroblasts^13,16^. Importantly, in both reprogramming systems, the resulting cells are equivalent to their in vitro blastocyst-derived counterparts in their transcriptome, epigenome, and function^13,15–17,20^.

While in the mouse system, nuclear reprogramming to pluripotency and TE state during fertilization or in nuclear transfer takes 2–3 days^21^, when done by transcription factors the process becomes inefficient^13,15–17^. Intrigued by these fundamental differences, scientists have devoted the last decade to monitoring and describing the various mechanisms, stages and pathways that underlie somatic cells undergoing reprogramming to pluripotency^14^. These major efforts have revealed key aspects in nuclear reprogramming which also explain, at least partially, the low efficiency of the process and describe in detail the trajectories somatic cells undergo in their way to become iPSCs. However, the characterization of the reprogramming process to the mouse TSC state has never been performed.

Here, we provide a multi-layer characterization of cells undergoing reprogramming to the TSC state, by conducting concomitant and comparative multi-omics analysis of cells acquiring both pluripotency and TSC state, which allowed us to also identify previously unknown properties for OSKM reprogramming. We show that in contrast to early embryonic cells, fibroblasts transduced with GETM or with OSKM mostly follow a ‘V’-shaped model where cells acquire, from the onset of the reprogramming process, a mostly mutually exclusive chromatin and transcriptional programs important for the induction of each state. This ‘V’- shaped behavior is also evident at the methylation levels, where correlation with transcription is relatively low. Single-cell analysis revealed previously unknown markers for each reprogramming system and a unique subpopulation within iTSCs with a transcriptome more similar to the TE compartment of the pre-implantation embryo. Chromatin accessibility and activity in conjunction with transcriptomic analyses identified global reprogramming blockers such as USF1/USF2, NRF2 and MAFK and TSC reprogramming facilitators such as TCF15.

The integrated data highlights key aspects of each fate. We show that from the onset, OSKM define regions that are developmentally important for the heart and brain, and, that while GETM shut off the embryonic program by DNA methylation, OSKM open these regions but retain them as inactive by eliminating the histone mark H3K27ac.

## Results

### Early embryogenesis follows a ‘T’-shaped progression

**Fig. 1:**
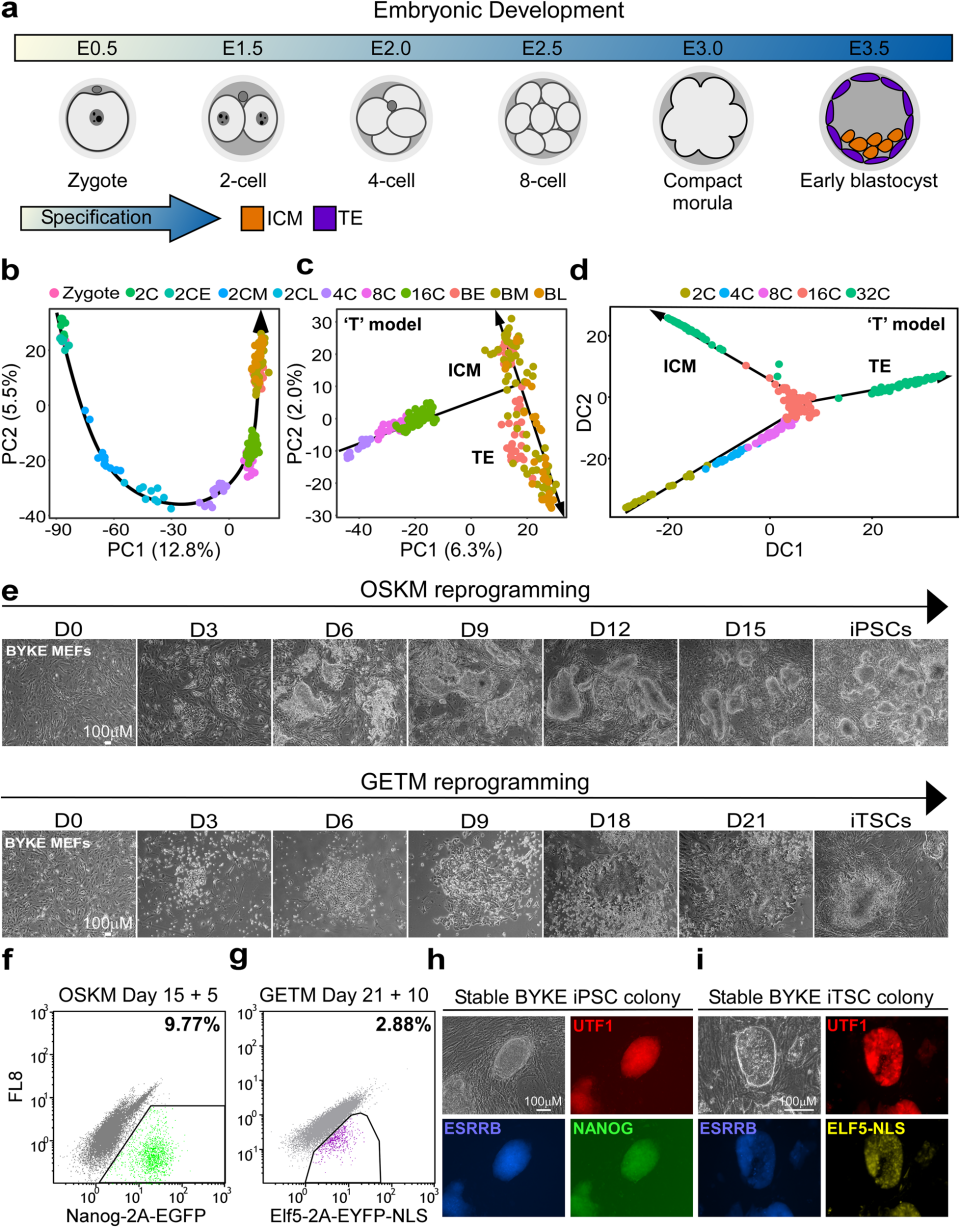
Establishment of the pluripotent and trophectoderm states in the embryo and during somatic nuclear reprogramming. **a** An illustration of early embryogenesis. Inner cell mass (ICM, orange) and trophectoderm (TE, purple) are the first compartments to show a clear transcriptional specification. **b**, **c** Single-cell RNA sequencing data obtained from different stages of developing embryo^5^ demonstrating the trajectory from zygote to blastocyst. PCA graphs showing gene expression profiles among 252 single cells. **b** The exclusion of totipotent cells (zygote and 2-cell stage (2 C)) allows the visualization of a T-like shape progression segregating the TE from the ICM. BE indicates blastocyst-early, BM indicates blastocyst-middle, and BL indicates blastocyst-late. **d** Single-cell Fluidigm BioMark analysis data obtained from different stages of developing embryo^7^ demonstrating the trajectory from the zygote to the blastocyst stage. Diffusion map was constructed by MERLoT package using 48 genes in 433 individual cells obtained from 2 C through blastocyst. **e** Representative bright field images showing cell morphology and cell density during OSKM reprogramming toward iPSCs (top) and during GETM reprogramming toward iTSCs (bottom). Eleven repetitions of independent reprogramming experiments were performed to collect the various samples for the multi-omics analysis (*n* = 11). **f**, **g** Representative flow cytometry analysis for Nanog-2A-EGFP (**f**) or Elf5-2A-EYFP (**g**) reporter on BYKE MEFs undergoing reprogramming for 15 days followed by 5 days of dox removal with OSKM factors (**f**) or for 21 days followed by 10 days of dox removal with GETM factors. Five independent reprogramming experiments were analyzed, all showing a comparable level of reporter activation (*n* = 5). For gating strategy, see Supplementary Fig. 9. **h** Representative bright field and fluorescence images of a stable BYKE iPSC colony demonstrating the activation of the three pluripotent reporters (Utf1-2A-tdTomato/Esrrb-2A-TagBFP/Nanog-2A-EGFP). Five colonies from five independent repetitions were analyzed, all showing the same signals (*n* = 5). **i** Representative bright field and fluorescence images of a stable BYKE iTSC colony demonstrating the activation of the three TSC reporters (Utf1-2A-tdTomato/Esrrb-2A-TagBFP/Elf5-2A-EYFP-NLS). Five colonies from five independent repetitions were analyzed, all showing the same signals (*n* = 5).

The trajectory from zygote to blastocyst (Fig. 1a) has been described by several studies using single-cell transcriptomic data^5–11^. Using principal component analysis (PCA), Deng et al. suggested that in the mouse, the trajectory follows a ‘U’ shape^5^, in which PC1 separates between the zygote/early 2-cell stage and blastocyst and PC2 separates between the other stages, namely the 2–16-cell stage and the zygote/blastocyst (Fig. 1b). However, since the zygote and the 2-cell stage are considered totipotent and thus harbor a unique transcriptome, we reanalyzed the data by excluding these two stages. Interestingly, the re-analyzed PCA revealed a clear ‘T’-like shape where PC1 separates between the four-cell stage and the blastocyst and PC2 between the ICM and TE (Fig. 1c, Supplementary Fig. 1a). More importantly, while both analyses (Fig. 1b, c) suggest that a clear transcriptional shift between different stages occurs during early embryogenesis, in both analyses the heterogeneity within each group was mild, indicating that the cells undergo relatively similar changes during embryogenesis and before specification. A ‘T’-shaped behavior of early embryonic cells was similarly observed in the datasets of Guo et al^7^., strengthening the notion that only at the morula/early blastocyst stage, a clear transcriptional segregation between cells of the same developmental stage can be witnessed (Fig. 1d, Supplementary Fig. 1b).

We sought to determine whether this ‘T’-like behavior also characterizes mouse somatic cells undergoing reprogramming to pluripotency and TSC state. In general, the reprogramming process of fibroblasts is characterized by multiple steps: (1) loss of somatic cell identity, (2) rapid proliferation, (3) mesenchymal to epithelial transition (MET), (4) metabolic shift, (5) stochastic gene expression, (6) epigenetic switch, (7) silencing of the exogenous factors by methylation, and finally (8) the stabilization of the core cellular circuitry^14,22^. We proposed three possible models, ‘T’, ‘Y’ and ‘V’, which may represent the trajectory of fibroblasts undergoing reprogramming into iPSCs and iTSCs (Supplementary Fig. 1c). The ‘T’-shaped model predicts that fibroblasts undergoing reprogramming into iPSCs or iTSCs will undergo comparable transcriptional and epigenetic changes during the conversion and that a separation between the two will occur only at the end of the reprogramming process, similarly to the cells of the early embryo. The ‘Y’-shaped model predicts that only genes and regulatory elements that are responsible for early and general processes like loss of somatic cell identity, proliferation and MET will be shared between the two systems, after which each will take a different path. The ‘V’-shaped model predicts that some early processes are shared by the two systems, but that each mostly employs a different set of genes and regulatory elements to achieve its own unique fate.

To understand which of the three proposed models most accurately represents the reprogramming process toward the two states, we performed a parallel, comparative, multi-omics analysis on fibroblasts undergoing reprogramming to iPSCs by OSKM or to iTSCs, by GETM factors simultaneously (Fig. 1e). We utilized our previously developed BYKE system for distinguishing between pluripotent and TSC reprogramming (Fig. 1f–i^23^,). We profiled the transcriptome (bulk RNA-seq and single-cell RNA-seq (scRNA-seq)), methylome (reduced representation bisulfite sequencing, RRBS), chromatin accessibility (ATAC-seq), chromatin activity (ChIP-seq for H3K4me2 and H3K27ac) and genomic stability (CNVs) at different time points along the reprogramming processes and ran computational analyses while comparing the behavior of the two systems (Supplementary Fig. 1d).

### Bulk transcriptomic dynamics for iTSC and iPSC reprogramming

**Fig. 2:**
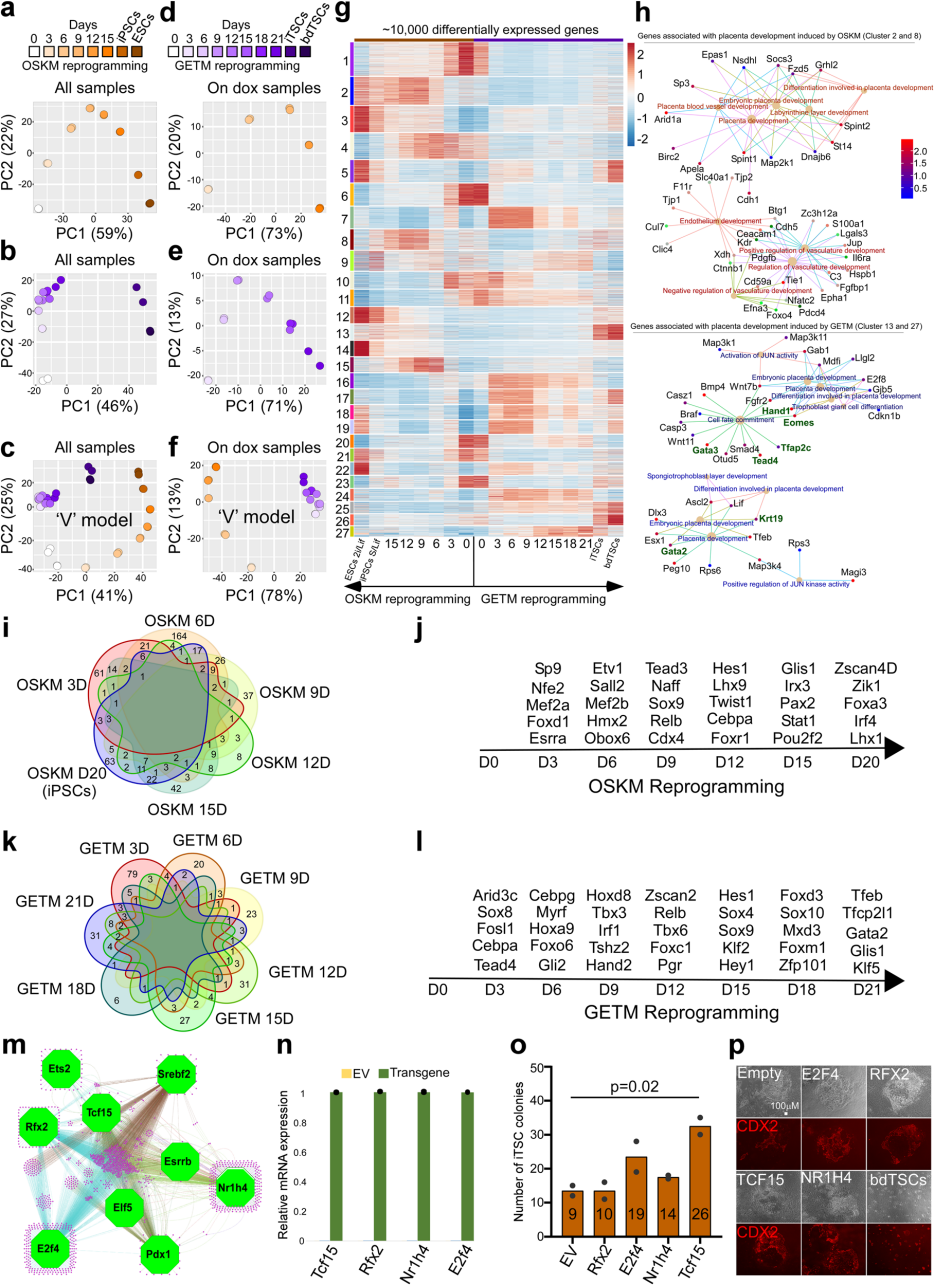
Bulk RNA-seq analysis on cells undergoing reprogramming to iPSCs and iTSCs. **a**–**c** PCA plots describing the trajectory during the reprogramming to either iPSCs (**a**), iTSCs (**b**) or both (**c**) as assessed by gene expression profiles (two biological replicates for each time point/sample, *n* = 2). **d**–**f** same as in (**a**–**c**) but here only induced cells (cells on dox) are plotted. **g** Heatmap of the most variable 10,000 genes among ESCs, bdTSCs, MEFs and cells during reprogramming. Unsupervised hierarchical clustering was performed and adaptive branch pruning was used to identify 27 prominent clusters. **h** Gene-concept network of GO terms associated with placenta development induced by OSKM (upper panel) or by GETM (lower panel) reprogramming. Key regulators of the TSC state are marked by green. **i**, **k** Venn diagrams plots for transcription factor dynamics during reprogramming with OSKM or with GETM, respectively (lfc > 1, p.adj ≤ 0.01). **j**, **l** Schematic illustrations showing representative transcription factors that are activated during the reprogramming to pluripotency or to TSC state, respectively. **m** A network generated with iRegulon Cytoscape plugin showing key transcription factors (green nodes) predicted to regulate 1288 genes (magenta nodes) that are completely silent or only mildly expressed in GETM day 21 but strongly expressed in iTSCs. **n**–**p** Oct4**-**GFP MEFs were transduced with GETM together with the indicated factor and reprogrammed for 20 days followed by 10 days of dox removal. **n** qPCR analysis of the indicated transgenes. The highest sample for each transgene was set to 1. Results were normalized to the *Gapdh* gene and are shown as fold change of two replicate runs in a typical experiment (*n* = 3). **o** Quantification of the number of stable iTSC colonies in the various reprogramming combinations. Numbers inside bars indicate the average number of CDX2-positive iTSC colonies of two independent biological replicate runs (*n* = 2). Asterisk indicates *p* value of 0.02 using two-tailed unpaired *t*-test calculated by GraphPad Prism (8.3.0). **p** Representative images for CDX2–positive (red) iTSC colonies in the indicated reprogramming combinations and bdTSCs from two independent experiments (*n* = 2). See also Supplementary Data file 1. Source data are provided as a Source Data file.

We used full-transcript RNA-seq to estimate expression levels and transcribed isoforms, with a total depth of 20 M reads per replicate. PCA exposed the trajectory cells undergo during reprogramming to iTSCs and iPSCs (Fig. 2a–f). We extracted the gene loadings associated with the first two principal components in each PCA plot to reveal those that drive the distinguishing stages/steps in and between the two reprogramming systems (Supplementary Fig. 1e–j). Notably, reprogramming to a TSC or pluripotent state exhibited a markedly different transcriptional landscape, and analyzing both processes together revealed a ‘V’-like shape, starting from the beginning of the process (Fig. 2c, f, Supplementary Figs. 1g, j), suggesting that major transcriptional changes separate the two systems.

While the reprogramming process toward iPSCs showed gradual transcriptional changes until stabilizing the final cells (Fig. 2a, d, Supplementary Fig. 1e, h), the process toward iTSCs showed two main waves of transcriptional change, the first occurring as early as day 3, PC2 (Fig. 2b, Supplementary Fig. 1f), followed by subtle transcription changes until day 21 (Fig. 2e, Supplementary Fig. 1i), whereupon after transgene expression removal a second wave is initiated, which is important for core TSC circuitry activation (i.e., PC1, Fig. 2b, Supplementary Fig. 1f).

These differences between OSKM and GETM reprogramming may be partially due to the nature of each reprogramming process. While iPSC colonies may be stabilized during reprogramming and in the presence of transgenes, iTSC colonies cannot. Only when transgenes expression is shut off (i.e., removal of dox) stable iTSC colonies emerge.

Next, the ~10,000 most differentially expressed genes were clustered into 27 unique clusters (Fig. 2g, Supplementary Data file 1). Clusters 1, 4, 6, 10, 11, 20, and 23 contain MEF-specific genes that are downregulated during GETM and OSKM reprogramming with unique dynamics for each cluster and system. Clusters 7, 16, 17, 18, 19, 24, 25, and 27 are specific to the TSC reprogramming process. Most clusters involve genes important for metabolism and cell cycle regulation. Clusters 2, 8, 12, and 15 are specific to iPSC reprogramming and contain genes that participate in cell junction organization, Ras protein signal transduction, and regulation of vasculature development. Clusters 3, 5, and 9 are shared between the two processes and composed of genes that regulate cell cycle, DNA repair, and Wnt signaling pathway (Supplementary Data file 1). Most genes behaved differently between the two reprogramming systems, even in early and shared dynamics such as proliferation, chromatin remodeling, and mesenchymal to epithelial transition (MET, Supplementary Fig. 2a–d, Supplementary Data file 1). While as expected, key mesenchymal genes and regulators of epithelial to mesenchymal transition are downregulated in both systems (Supplementary Fig. 2c, bottom of the heatmap, Supplementary Data file 1), indicating loss of fibroblastic identity, particular mesenchymal and MET-specific genes are uniquely expressed in each reprogramming system (Supplementary Fig. 2c, d). Another example of an important difference between the two systems is observed already in early reprogramming stages is a metabolic shift occurring in two waves (days 3–9 and 12–21) in iTSC reprogramming. This shift, which plays a role in translation regulation and RNA processing, is absent in iPSC reprogramming (Supplementary Fig. 2e–g, Supplementary Data file 1). Moreover, even when similar GO terms are annotated between the two systems, each reprogramming system utilizes different sets of genes to execute the process. Figure 2h shows an example where both iTSC and iPSC reprogramming systems activate placenta/trophoblast- specific genes with ‘placenta development’ GO annotation. However, while OSKM activate trophoblast differentiation genes, GETM activate trophoblast stem cell genes (marked by green, Fig. 2h, Supplementary Data file 1). Pursuant to this, each conversion system exhibits a unique set of key transcription factors along the reprogramming process, suggesting a ‘V’-shaped behavior (Fig. 2i–l).

To improve the efficiency of the reprogramming process to iTSCs, we extracted from the bulk RNA-seq data a list of 1288 genes that are silent or only mildly expressed in GETM day 21 but strongly expressed in iTSCs. We postulated that if these genes are essential for the stabilization of the TSC core circuitry, activating them earlier will increase reprogramming efficiency. Using iRegulon^24^ we identified nine potential regulators for this gene set: RFX2, TCF15, E2F4, ESRRB, ELF5, ETS2, NR1H4, PDX1, SREBF2. Of those, ELF5, ETS2, and ESRRB have been shown to increase the reprogramming process to iTSCs^13,16,25^. Cloning 4 of the others, *E2f4*, *Tcf15*, *Rfx2*, and *Nr1h4*, into dox-inducible lentiviral vectors, showed that *Tcf15* and to a lesser extent *E2f4* and *Nr1h4* increase reprogramming efficiency toward iTSCs (Fig. 2m–p).

Thus, stepwise bulk transcriptomic analyses identified new iTSC reprogramming facilitators and propose a clear ‘V’-shaped behavior between GETM and OSKM whereby each reprogramming system operates distinctively to reprogram the somatic nucleus.

### Single-cell transcriptomics for iTSC and iPSC establishment

Typically, of the cells destined for iTSC or iPSC reprogramming, only 1–5% and 10–20% respectively acquire the fully reprogrammed state^13,23^ (Fig. 1f, g). Although powerful, bulk transcriptional analysis lacks the sensitivity to identify such small fractions of cells. Population averaging likewise ambiguates a ‘T’ or ‘Y’ transcriptional behavior among small groups of cells that harbor two reprogramming systems. We therefore conducted single-cell analysis on GETM and OSKM induced cells.

**Fig. 3:**
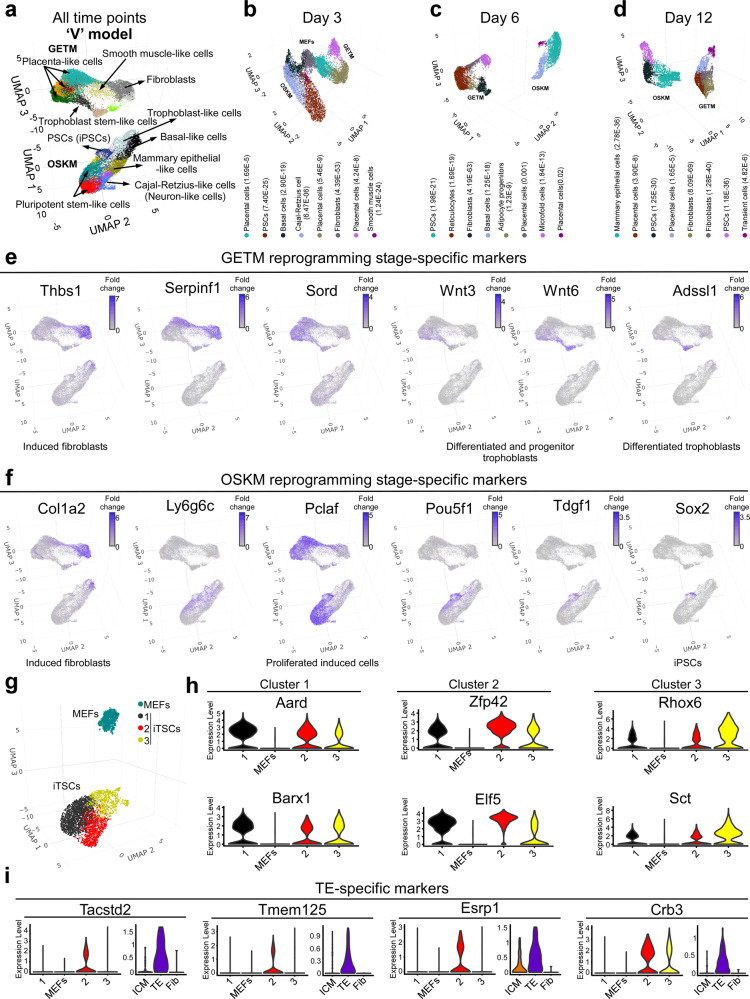
Single-cell RNA-seq analysis separates OSKM from GETM reprogramming. **a** Uniform Manifold Approximation and Projection (UMAP) visualization analysis of 23,446 cells at days 3, 6, and 12 during OSKM and GETM reprogramming. Each point represents a single cell and each color represents a unique community among the population. The most significant cell type was assigned to each cluster of cells using EnrichR- PanglaoDB Augmented 2021. **b**–**d** UMAP visualization of 9708 single cells profiled at day 3 (**b**), 7806 single cells at day 6 (**c**), or 5932 single cells at day 12 (**d**) of both OSKM and GETM reprogramming. Each point represents a single cell and each color represents a unique community among the population. The most significant cell type was assigned to each cluster of cells using EnrichR- PanglaoDB Augmented 2021. **e**, **f** Expression level of selected cluster-specific markers for GETM (**e**) and OSKM (**f**) reprogramming, respectively. The expression level of the specified markers is visualized in cells within the UMAP by a range of intensities of a purple color. **g** UMAP visualization of 3393 single iTSCs and parental MEFs. Three clusters were identified showing a significant heterogeneity within the iTSCs cells. **h** Violin plots summarizing single-cell expression level of specific marker genes for each cluster of iTSCs (p.adj ≤ 0.05). **i** Violin plots showing prevalent expression-specific markers at the single-cell level for genes shared between cells within cluster 2 and the TE compartment of the embryo^5^. See also Supplementary Data file 2.

To evaluate the transcriptomes of individual cells undergoing reprogramming into iPSCs and iTSCs, we harnessed the 10× Genomics platform and profiled the transcriptome of 26,839 single cells at days 3, 6, and 12 along the reprogramming processes as well as the transcriptome of stable iTSCs. These timepoints represent the stochastic gene expression phase in the two reprogramming systems, when the highest variation between individual cells in expected. UMAP analysis for these timepoints demonstrated two distinct cells clusters, one for GETM and one for OSKM reprogramming, suggesting a V-like shaped behavior (Fig. 3a). In each timepoint the only overlapping cells between the two reprogramming systems are the parental MEFs that are still present at day 3 of reprogramming (Fig. 3b–d). Using EnrichR^26^ “PanglaoDB Augmented 2021” different transcriptional fates for induced cells were identified during each reprogramming process, with both containing cells with a transcriptional profile partially similar to that of the placenta, in accordance with the bulk analysis. Other than fibroblasts and placental cells, different groups of induced cells activated gene signatures that are enriched in Basal cells, Cajal-Retzius cells and mammary epithelial cells in OSKM reprogramming and Reticulocytes, Adipose progenitors, Microfold cells and Transient cells in GETM reprogramming (Fig. 3a–d, Supplementary Data file 2). Epidermis, placenta and neuronal fates have previously been observed in OSKM reprogramming, strengthening our findings^27^. We next identified known and unknown stage-specific markers for each reprogramming process (Fig. 3e, f). For iTSC reprogramming, non-infected MEFs or refractory MEFs were identified using the known mesenchymal markers *Col1a2* and *Thbs1*. *Serpinf1* marks both MEFs and cells that have succeeded to initiate the reprogramming process. *Sord* marks most induced cells that are in the midst of the reprogramming process prior to any fate decision while *Wnt3*, *Wnt6* and *Adssl1* mark cells that are either differentiated trophoblasts or those that are probably destined to become iTSCs, based on their unique intermediate stemness gene signature (Fig. 3e).

For OSKM reprogramming, *Ly6g6c* represents cells that underwent MET and have acquired transcriptional profile of either placenta or epidermal fate. *Pclaf* marks those cells that are highly proliferative and are in the midst of the reprogramming process of both reprogramming systems. *Tdgf1* represents a successful trajectory to reprogramming based on gene expression (Fig. 3f). Finally, as we and others have previously noted^14,28,29^, while *Oct4* fails to mark fully reprogrammed cells, *Sox2* stringently mark them (Fig. 3f).

We next used both the bulk RNA-seq and the scRNA-seq data to identify genes that robustly distinguish between GETM-induced and OSKM-induced cells. While *Arhgdib*, *Id3*, *Tm4sf1*, *Egfl7*, *Plac1*, *Prl8a9*, and *Slc38a3* mark specifically GETM-induced cells, *Shisa8*, *Fetub*, *Slc7a3*, *Tdh*, *Nccrp1*, *Ehf*, and *Krt17* are uniquely expressed in OSKM-induced cells (Supplementary Fig. 3a–d). Interestingly, the proliferation rate of the two systems was different as well, while both contained Myc. Based on the expression of proliferation gene signature, OSKM-induced cells proliferate faster than GETM-induced cells, while non-induced cells from both systems expressed these genes at a very low level (Supplementary Fig. 3e).

We next analyzed the transcriptomic landscape of stable iTSCs. Interestingly, although the cells were grown in a defined TSC medium (TX) that maintains a homogenous stemness morphology^30^, we observed a significant heterogeneity within individual cells of the same colony. UMAP analysis for the parental MEFs and the resulting iTSCs revealed three major clusters for iTSCs (Fig. 3g). While both cluster 1 and 2 contain cells with stemness signature, cluster 2 expresses high levels of TSC key master regulators such as *Elf5*, *Zfp42* (Fig. 3h), *Eomes*, *Utf1*, *Epcam*, and *Gata3*, and cluster 1 expresses high levels of other stemness gene such as *Aard* and *Barx1* (Fig. 3h). In contract, cluster 3 represents cells that start to exit the TSC state and express low levels of stemness genes and higher level of differentiation genes such as *Rhox6* and *Sct* (Fig. 3h). Interestingly, a low number of cells in cluster 2 show unique expression of TE-specific genes such as *Tacstd2*, *Tmem125*, *Esrp1*, *Crb3*, suggesting that within an iTSCs population a small fraction of cells is more similar to the TE compartment of the pre-implementation blastocyst (Fig. 3i, Supplementary Fig. 3f, g), an observation that may explain the low contribution capability of TSCs/iTSCs to developing placenta. Overall, these results illuminate the various fates, stage-specific markers and unique identifiers for each reprogramming system and stable iTSCs and strengthen the notion that each reprogramming process takes a distinct trajectory toward its own fate from the onset of the reprogramming process.

### GETM and OSKM reprogramming methylation dynamics

A crucial aspect of nuclear reprogramming is erasure of the epigenetic landscape of the somatic nucleus. An important epigenetic mark of which is DNA methylation, that allows chromatin condensation and silencing of specific loci^14^. To assess the methylation landscape of somatic cells undergoing reprogramming to iTSCs and iPSCs, we applied the RRBS technique to capture the CpG methylation landscape as a representation for the global methylation changes. GETM and OSKM-induced cells from different timepoints (Supplementary Fig. 1d), and parental fibroblasts, bdTSCs, iTSCs, ESCs, and iPSCs, were subjected to RRBS.

**Fig. 4:**
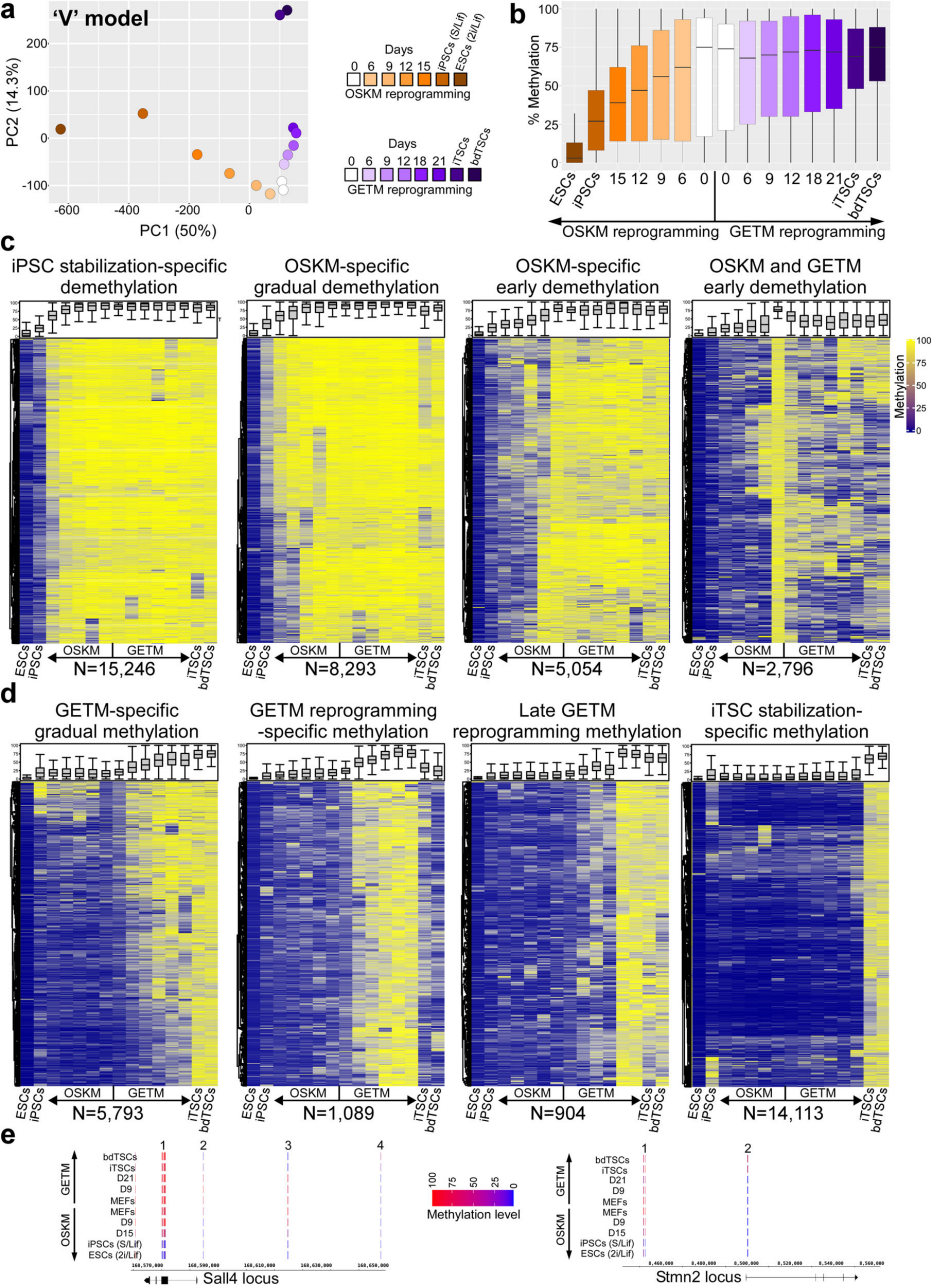
RRBS analysis demonstrates methylation specific dynamics between OSKM and GETM reprogramming. **a** Average bulk DNA methylation data of cells undergoing reprogramming toward pluripotency and TSC state projected onto the first two principal components. A clear V-like shape progression is observed separating GETM from OSKM reprogramming. **b** Boxplot of overall DNA methylation level across the indicated bulk samples (for each ample and time point two biologically independent replicates were analyzed, *n* = 130,142) during reprogramming toward both pluripotent and TSC states. Boxes indicate 50% (25–75%) and whiskers (5–95%) of all measurements, with black lines depicting the medians. **c**–**d** Heatmaps demonstrating the dynamics of DNA methylation alterations and patterns across bulk samples during reprogramming toward both pluripotent and TSC states, respectively. Each row represents one differentially methylated block for which there are at least one CpG with ≥10× coverage. Boxplots at the top of each heatmap depict the DNA methylation level across the indicated bulk samples (for each sample and time point two biologically independent replicates were analyzed, (**c**) *n* = from left to right: 15,246, 8293, 5054, 2796; (**d**) *n* = from left to right: 5793, 1089, 904, 14,113) during reprogramming toward both pluripotent and TSC states. Boxes indicate 50% (25–75%) and whiskers (5–95%) of all measurements, with black lines depicting the medians. **e** Genome browser snapshot showing RRBS-captured CpG sites (short blue, purple or red lines) of the indicated samples in *TSC*-*ESC-shared loci* (i.e., *Sall4* and *Stmn2*). Scale bar indicates methylation levels ranging from no methylation (blue), intermediate methylation (purple) to maximum methylation (red). See also Supplementary Data file 3.

We used the K-means algorithm to classify ~130,000 genomic regions (blocks) shared amongst all samples during reprogramming to a TSC or pluripotent state, and generated 100 unique clusters, some containing tiles that are specific to TSCs and ESCs, others to MEFs and the vast majority to GETM- and OSKM-induced cells (Supplementary Fig. 4a). Average DNA methylation levels per sample per cluster were then projected onto the first two principal components, generating gradual, time-dependent methylation dynamics for each reprogramming system with a clear ‘V’-shaped trajectory, where PC1 represents the OSKM trajectory and PC2 the GETM trajectory (Fig. 4a). The accuracy of the time-dependent methylation trajectory in the two reprogramming systems was surprising, given the often poor correlation between methylation degree and gene activation, and the unique transcriptional profiles that characterize intermediate induced cells (Fig. 2d, e).

One clear difference between OSKM and GETM reprogramming was the overall dynamic of methylation changes during the reprogramming process (Fig. 4b). While OSKM-induced cells predominately lose methylation on CpG-enriched sites during the reprogramming process, GETM-induced cells mostly gain methylation on CpG-enriched sites either in the middle or gradually until the end of the reprogramming process (Fig. 4b–d, Supplementary Fig. 4b–d).

Mammalian placentas are unique in their methylation landscape as they contain regions in the genome that are highly methylated in gene bodies and regions that are only intermediately methylated (40–60%)^31^. In accordance with that, our unbiased analysis identified two unique clusters that contain intermediately methylated regions only in the final and stabilized iTSCs/TSCs (Supplementary Fig. 4e). These results suggest a unique mechanism of methylation/demethylation that occurs only when the core TSC circuitry is activated.

We associated the clusters’ tiles to their neighboring genes and performed GO analysis using GREAT^32^ (Supplementary Data file 3). While clusters associated with gradual loss of methylation in OSKM reprogramming include genes involved in maintenance of fibroblastic identity, apoptosis, and multiple somatic cell properties, the single cluster that exhibits early demethylation in both systems is composed of genes that participate in somatic stem cell maintenance, immune system development and regulation of growth (Supplementary Data file 3). While demethylation of regions related to stemness and growth is expected in the two systems, the identification in both systems of a set of genes enriched in the immune system, in methylation pattern and RNA, is intriguing (Supplementary Fig. 3a, b, Supplementary Data file 3).

Clusters associated with a gradual gain of methylation specifically in GETM reprogramming mostly include genes that negatively regulate metabolic processes of RNA production and transcription. In accordance with their identity as extra-embryonic cells, clusters that involve methylation only at the final step of the reprogramming process and in the fully reprogrammed cells comprise genes that are essential for embryo development, neuronal lineage development and somatic cell differentiation at large (Supplementary Data file 3). Thus, while GETM factors utilize methylation to disable master genes important for executing the embryonic development program, OSKM first open these regions and subsequently regulate their expression by histone modifications (as discussed in the next section). Of special note is the neuronal lineage: while OSKM activate this program, GETM induce its silencing.

Since pluripotent cells and TSCs share many stemness genes (e.g., *Sall4*, *Esrrb*, *Sox2*, *Lin28* etc^13,23^.), we next asked whether we can identify methylation differences in their regulatory elements during reprogramming and in the final cells. We selected 6 genomic *loci* that contain tiles for genes that are either specific to pluripotent cells (*Slc15a1* and *Tex19.2*), specific to TSCs (*Eomes* and *Bmp8b*), or shared between the two cell types (*Sall4* and *Stmn2*). Interestingly, only few tiles on regulatory elements (e.g., tile block number 2 and 3 in *Sall4 locus*) were methylated/hypomethylated similarly between iPSCs/ESCs and bdTSCs/iTSCs and different from MEFs, weakening the notion of widespread shared regulatory elements between the two cell types (Fig. 4e). Most tiles on regulatory elements were methylated oppositely between the two cell states (Fig. 4e, Supplementary Fig. 4f, g), indicating tight and cell type-specific regulation for each reprogramming process.

Taken together, these data suggest that although the acquisition of the final methylation landscape of GETM and OSKM induced cells is gradual and time-dependent, the methylation level and deposition is unique for each reprogramming process, even in genes that are expressed in both cell types.

### Chromatin dynamics during iTSC and iPSC establishment

One of the properties of reprogramming factors is their ability to open closed chromatin by recruiting chromatin remodelers and transcriptional machinery to heterochromatin^33,34^. Chromatin accessibility and activity can be assessed by an Assay for Transposase-Accessible Chromatin using sequencing (ATAC-seq)^35^ coupled with chromatin immunoprecipitation and sequencing (ChIP-seq) for specific histone marks. Using these assays, we compared cells collected at day 3, 6, and 9 of the reprogramming process to iTSCs and iPSCs, their parental MEFs, the final reprogrammed cells and their blastocyst-derived controls. We chose H3K27ac and H3K4me2, because H3K27ac marks both active promoters and distal enhancers, while H3K4me2 marks genes primed for future expression^36,37^, and is also enriched in *cis*-regulatory regions of transcriptionally active genes, particularly in promoters.

Overall, we analyzed 170,658 peaks for ATAC-seq, 498,376 for H3K27ac and 770,274 for H3K4me2. This allowed us to map in each reprogramming system the regions that are open and active early on, those primed to be active later, and closed regions.

**Fig. 5:**
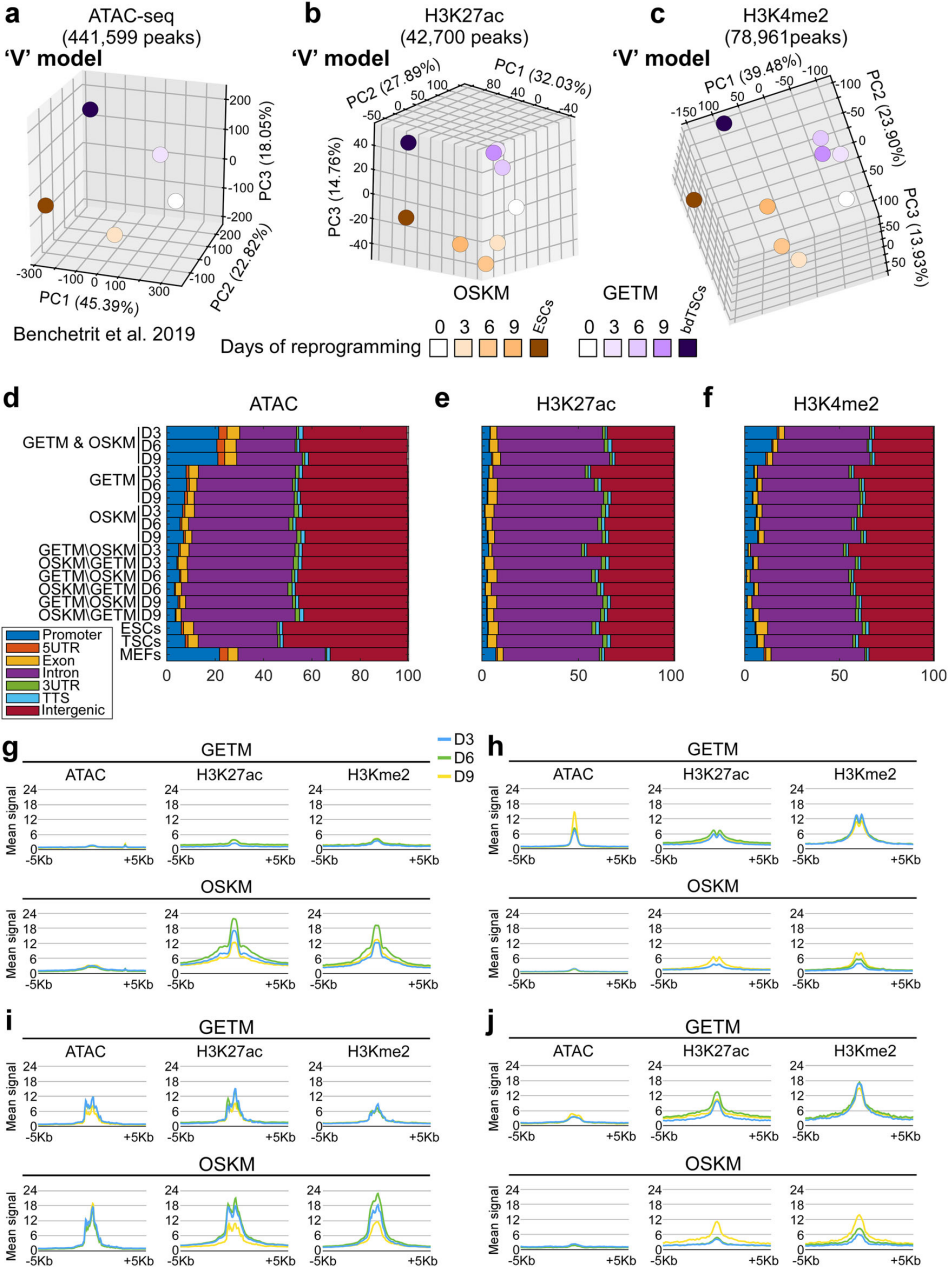
Chromatic accessibility and activity during GETM and OSKM reprogramming demonstrating a ‘V’-shaped behavior. **a**–**c** Top 3 PCA components of the *Z*-scores of ATAC-seq (**a**), H3K27ac (**b**) and H3K4me2 peaks (**c**). Peaks were clipped to range [0, 500] and filtered by length (≥500 bp). Replicates were merged by taking the mean peak height. **d**–**f** Genomic annotations of ATAC-seq peaks (**d**), H3K27ac peaks (**e**) and H3K4me2 peaks (**f**). Shown are the fraction of various genomic annotations (Promoter, Exons, Introns, etc) among peaks. Genomic regions accessible in both GETM and OSKM conditions (**d**, top three rows) are enriched for promoter regions, compared to GETM or OSKM regions (below). GETM and OSKM mark regions accessible in those conditions, excluding MEF peaks. Below are cell-type specific accessible regions such as “GETM\OSKM D3”, which includes GETM Day 3 peaks not accessible in OSKM Day 3. In addition to Promoter regions (blue), most accessible regions fall within Intronic regions (purple) and Intergenic regions (red). **g**–**j** Mean ATAC-seq, H3K27ac and H3K4me2 at ±5Kb surrounding OSKM or GETM H3K27ac peak locations, for Day 3 (blue), Day 6 (green), and Day 9 (yellow). **g** OSKM-specific H3K27ac signal is strongest at Day 6, and is accompanied by matching H3K4me2 signal, but with no dynamic change in DNA accessibility. **h** Same for GETM ATAC-seq peaks. These regions are already marked by H3K4me2 in Day 3, and gain accessibility over time. These genome regions also show H3K27ac and H3K4me2 enrichments for later OSKM stages. **i** Same for OSKM ATAC-seq peaks. **j** Same of GETM H3K27ac peaks. These peaks show gradual increase in ChIP-seq signal even following OSKM induction (below).

As the transcriptome and methylome results, PCA on datasets of chromatin accessibility (ATAC-seq) and activity (H3K27ac and H3K4me2) revealed two separate ‘V’-shaped trajectories distinguishing OSKM from GETM reprogramming, already at the process onset (Fig. 5a–c), suggesting that OSKM and GETM remodel the chromatin at different regions. An examination of the distribution of the peaks showed that while their location along the genome (i.e., promoters, exons, introns, UTRs, TSS and intergenic) is mostly different between the two reprogramming systems (Supplementary Fig. 5a–c), their distribution is very similar (Fig. 5d–f).

While shared OSKM and GETM ATAC-seq peaks and, to a lesser extent H3K4me2 peaks, exhibit a significant enrichment in promoters and exons, peaks that are unique to each reprogramming process are mostly localized to introns and intergenic regions (Fig. 5d and f). EnrichR GO analysis on the genes associated with the shared open promoters suggested that many of these active promoters are associated with response to the lentiviral infection itself (*p* ≤ 0.001). No significant differences in the distribution of H3K27ac was found between the various samples. These data suggest that the reprogramming process follows the same rules of genomic remodeling, i.e., it begins with robust opening of intronic and intergenic regions in conjunction with promoter closing, but that each reprogramming system remodels the chromatin at different loci along the genome in accordance with its final cellular fate.

By classifying the peaks (mean ATAC-seq, H3K27ac and H3K4me2 at ±5Kb) based on their behavior in the reprogramming systems (Supplementary Fig. 6a–d), we identified four distinct patterns: (1) 1605 genomic regions appearing predominantly in OSKM reprogramming. The H3K27ac signal of these regions was typically the strongest at day 6, and accompanied by a matching H3K4me2 signal, but with no dynamic change in DNA accessibility (Fig. 5g). (2) 1716 GETM-specific regions marked by H3K4me2 and H3k27ac already at day 3 but with chromatin accessibility gained only later in the process (i.e., day 9). Intriguingly, a mirror image can be seen in these regions during OSKM reprogramming. There, chromatin accessibility is mildly open and remains unchanged but a significant increase in H3K27ac and H3K4me2 signals is observed at later stages of reprogramming (Fig. 5h). (3) 2848 regions that are open and active in both reprogramming systems but lose activity (i.e., H3K27ac and H3K4me2 signal) over time exclusively in OSKM reprogramming (Fig. 5i). (4) 464 regions that are open in both reprogramming processes, but while in GETM reprogramming they are active throughout the process, in OSKM reprogramming they gain activity exclusively at later stages (Fig. 5j).

GREAT showed that group 1 is associated with cellular response to leukemia inhibitory factor (*p* ≤ 8.12e−23), Ras guanyl-nucleotide exchange factor activity (*p* ≤ 5.0e−9) and a mouse phenotype of embryonic lethality between implantation and placentation (*p* ≤ 1.5e−12). Group 2 was associated with cell migration and motility (*p* ≤ 2.6e−20), cell adhesion (*p* ≤ 4e−14), extracellular matrix (*p* ≤ 1.0e−8), insulin-like growth factor binding (*p* ≤ 2.7e−8) and heparin binding (*p* ≤ 3.0e−7), all relevant to trophoblast differentiation and placentation. Interestingly, once again, this group of genes is significantly enriched in cells of the immune system, giving rise to the GO term of mouse phenotype of autoimmune response (*p* ≤ 3.4e−13). These results suggest a mechanism by which GETM induce a TSC fate by gradually opening and activating trophoblast-specific regions that are important for TSC function. In contrast, OSKM do not change the accessibility of these regions, which remain mildly open, but then gradually activate them, which explains the small fraction of differentiated trophoblast cells present in OSKM reprogramming.

Group 3 regions are related to signaling pathways of platelet-derived growth factor receptor (*p* ≤ 1.8e−5), ERBB (*p* ≤ 8.5e−5), epidermal growth factor receptor (*p* ≤ 1.5e−4), with a mouse phenotype of placental labyrinth hypoplasia (*p* ≤ 2.6e−5). Group 4 genes involve cell motility and migration (*p* ≤ 8.1e−8), focal adhesion (*p* ≤ 1.3e−14) and actin cytoskeleton (*p* ≤ 3.6e−13).

Overall, OSKM and GETM factors open and activate regions that are essential for their function as well as regions that are important for the induction of MET and cellular transformation.

**Fig. 6:**
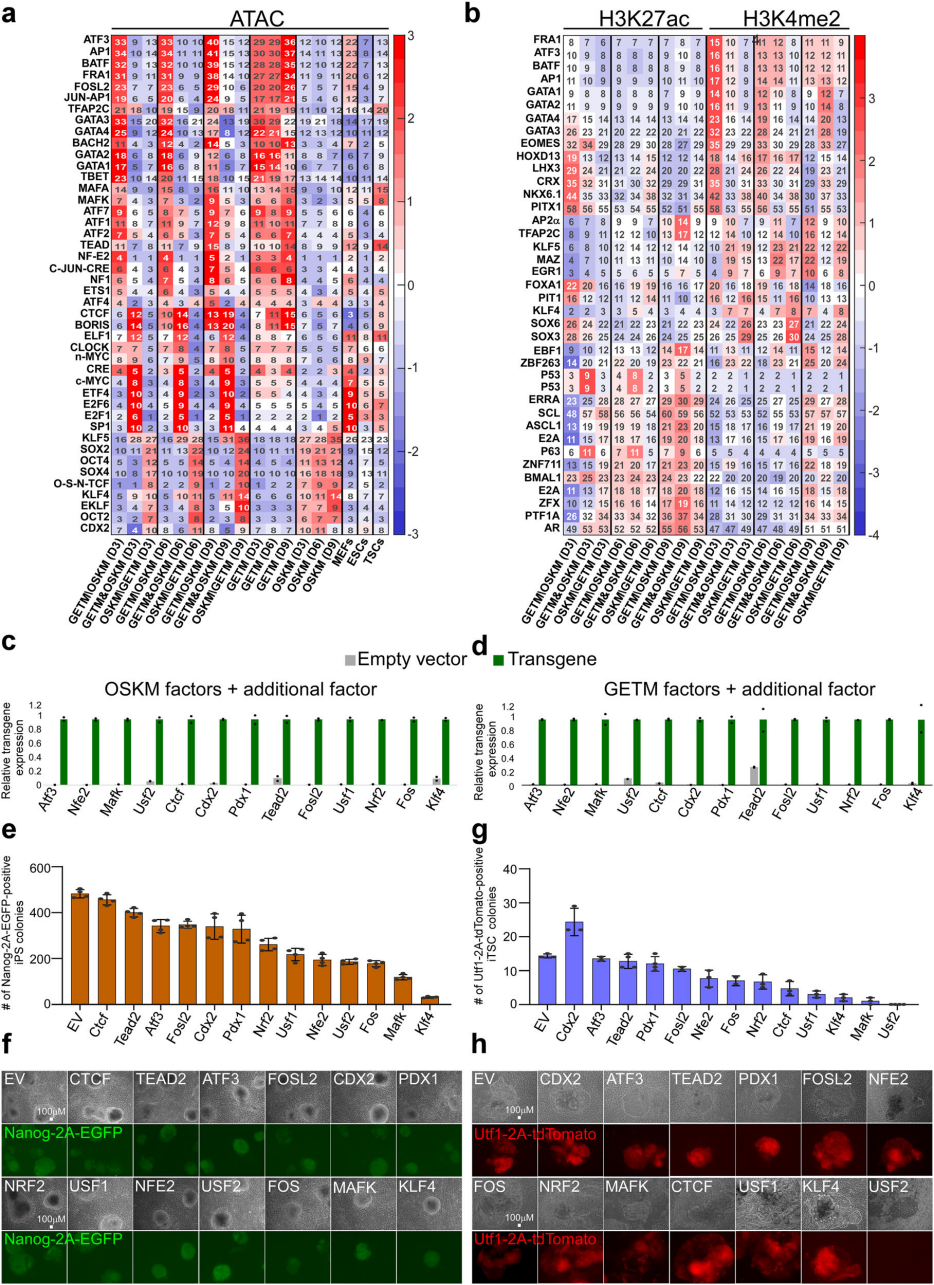
Motif enrichment and the effect of their corresponding transcription factor on OSKM and GETM reprogramming. **a** Heatmap showing motif enrichment among ATAC-seq peaks. For each row (motif) and each column (condition-specific ATAC-seq peaks) we calculated the percent of peaks containing it (shown numbers). Subsets of peaks include GETM-only peaks (GETM\OSKM), joined peaks (GETM&OSKM), and OSKM-only peaks (OSKM\GETM) for each time point (Days 3, 6, 9). Also shown are joined sets of GETM and OSKM peaks for each day, as well as MEF, ESC and TSC peaks. Each motif/condition is color-coded based on relative motif enrichment (*Z*-scores) compared to all conditions. Only motifs with enrichment greater than 2.5 standard deviations (*Z* > 2.5) are shown. **b** Heatmap showing motif enrichment among H3K27ac and H3K4me2 peaks. For each row and each column, we calculated the percent of peaks containing it (shown numbers). Subsets of peaks include GETM-only peaks (GETM\OSKM), joined peaks (GETM&OSKM), and OSKM-only peaks (OSKM\GETM) for each time point (Days 3, 6, 9). **c**, **d** BYKE MEFs were infected with dox-inducible OKSM STEMCCA cassette (**c**) or GETM factors (**d**) plus additional factor as depicted. qPCR analysis for the indicated transgenes is shown. The highest sample for each transgene was set to 1. Results were normalized to the *Gapdh* gene and are shown as fold change of two replicate runs in a typical experiment (*n* = 3). **e**, **f** BYKE MEFs were reprogrammed with OSKM together with the indicated factor for 8 days and then weaned of dox for additional 5 days. Nanog-2A-EGFP-positive colonies were counted (**e**) and imaged (**f**) in four independent reprogramming experiments (*n* = 4). EV refers to empty vector control. Error bars presented as a mean ± standard deviation of four replicates. **g**, **h** BYKE MEFs were reprogrammed with GETM together with the indicated factor for 21 days and then weaned of dox for additional 10 days. Utf1-2A-tdTomato-positive colonies were counted (**g**) and imaged (**h**) in four independent reprogramming experiments. Error bars presented as a mean ± standard deviation of 2–4 replicates. EV refers to empty vector control. Source data are provided as a Source Data file.

Next, we subtracted all the peaks that were overlapped with MEFs to identify transcription factor binding sites that are enriched in peaks (ATAC-seq, H3K27ac and H3K4me2) associated with each reprogramming process at various reprogramming time points. (Fig. 6a, b, Supplementary Figs. 5a–c, 6e, f). We observed highly significant *P* values for binding motifs of OSK factors in OSKM reprogramming peaks and GET binding motifs in GETM reprogramming peaks, supporting our analysis (Fig. 6a, b, Supplementary Figs. 5a–c, 6e, f). Binding sites of the AP1/CREB/ATF protein families, which act as somatic cell identity safeguards that block reprogramming to pluripotency^38^, are significantly more enriched in GETM compared to OSKM reprogramming peaks (Fig. 6a, b, Supplementary Fig. 5a–c). In contrast, open regions of fibroblasts that close upon reprogramming, forming the binding sites of the AP1/CREB/ATF family of proteins are significantly more enriched in OSKM compared to GETM reprogramming (Supplementary Fig. 6g, h). These results explain the potent ability of OSKM to erase somatic cell identity, as well as the presence of MEF-like cells in GETM reprogramming even at day 12 (Fig. 3a–d). In addition to GATA, TFAP2C and EOMES/TBET motifs, GETM-specific peaks are enriched with binding site of factors involved in oxidative stress response such as NRF2^39^, NFE2^39^, MAFK^39^ and BACH1/2^40^ (Fig. 6a, b, Supplementary Figs. 5a–c, 6e). In contrast, OSKM-specific peaks are enriched with pluripotency binding sites such as KLF, SOX, OCT and NANOG as expected, but also with binding sites of factors involved in neuronal differentiation, such as E2A^41^, ASCL1^42^, and with trophoblast such as CDX2 and ZNF263 (Fig. 6a, b, Supplementary Figs. 5a–c, 6f), explaining the generation of trophoblast-like cells and neuronal fate observable in OSKM reprogramming (Fig. 2h, 3b). In agreement with their role as reprogramming factors, GETM and OSKM shared peaks are enriched with motifs of proteins that are important for chromatin remodeling such as CTCF^43^, BORIS^44^, E2F6^45^, ELF1^46^, USF1/2^47^ and YY1^48^.

We performed reprogramming experiments to iPSCs and iTSCs with BYKE cells transduced with either OSKM or GETM, together with an empty vector (EV) control or with one of 13 selected factors (Fig. 6c, d), whose binding sites were significantly enriched in either GETM reprogramming (NRF2, NFE2, FOS, MAFK, ATF3, FOSL2, TEAD2) or in OSKM reprogramming (KLF4, CDX2, PDX1) or in both (CTCF, USF1, USF2, Fig. 6a, b). All factors besides CDX2 in GETM, either hindered the reprogramming process, or had a mild effect in both systems, suggesting that both OSKM and GETM initially open regions that are highly regulated by somatic identity safeguards, that counteract the reprogramming globally (Fig. 6e–h).

A strong reprogramming inhibition was noted when *Klf4* was overexpressed in both GETM and OSKM reprogramming. As *Klf4* is relatively highly expressed in MEFs, its overexpression on top of OSKM may be hypothesized to alter the stoichiometry of the reprogramming factors, counteracting reprogramming by maintaining fibroblastic identity. Another strong reprogramming blocker that we found, especially for iTSC reprogramming, is USF2, a known tumor suppressor and MYC inhibitor^49^. USF2 is a strong regulator of iron metabolism and oxidative stress response^50,51^, possibly explaining its stronger effect on iTSC reprogramming (Fig. 6e–h).

In contrast to the global reprogramming blockers mentioned above, CTCF, which significantly hindered the reprogramming to iTSCs, only mildly affected the reprogramming to iPSCs (Fig. 6e–h). As *Ctcf* is highly expressed in iTSCs/TSCs and acts as an important chromatin insulator that controls gene expression^52^, this result emphasizes the importance of retaining normal levels of CTCF for the induction of the TSC fate.

We then analyzed the binding sites of closed regions; peaks that were open in MEFs and disappeared during the reprogramming process with GETM or OSKM (Supplementary Fig. 6g, h). While OSKM closed peaks are enriched with binding sites for AP1/CRE/ATF family, TEAD, PDX1, RUNX and MEF2A, indicating the initial loss of the fibroblast identity, GETM closed peaks are enriched as well with binding sites for RUNX and TEAD but also with interferon response factors such as STAT5, ISRE, RXR and IRF1/2 and apoptosis-related genes such as P53 and P63. This might suggest that GETM overcome viral infection-induced apoptosis by closing regions that control interferon response genes and master regulators of cell death.

To determine whether regions that begin to open via GETM and OSKM, are active during the initial reprogramming phase, we probed using scatter plots all ATAC-seq peaks from GETM and OSKM reprogramming (Supplementary Fig. 7a). In accordance with the PCAs and Venn diagrams, the vast majority of peaks are unique to each reprogramming process. We then plotted all the H3K4me2 peaks on top of the ATAC-seq peaks (Supplementary Fig. 7b) and performed GO analysis on OSKM or GETM-specific peaks using EnrichR (Supplementary Fig. 7c, d). By comparing OSKM and GETM H3K4me2 peaks, we were able to focus on all the unique regions that are remodeled by OSKM and by GETM, as any global region involved in the identity of the fibroblast or important for reprogramming at large, will overlap between the two reprogramming systems. Remarkably, besides regions involved in the regulation of epithelial cell migration, analyzing OSKM-specific peaks revealed significant enrichment for regions important for the development of the heart, the first and arguably most crucial organ to form during embryogenesis (Supplementary Fig. 7c). Moreover, a significant enrichment was found for the formation of the brain and liver as well (Supplementary Fig. 7c). In contrast, GETM-specific H3K4me2 peaks are enriched for regions that involve metabolic processes and proliferation, as well as regions that are essential for trophoblast function such as migration and attraction of blood vessels (Supplementary Fig. 7d).

We then plotted the active histone mark H3K27ac on top of the ATAC-seq peaks (Supplementary Fig. 7e). We noted that while OSKM-specific ATAC-seq peaks tend to lose H3K27ac during the reprogramming process, GETM-specific peaks gain H3K27ac (Supplementary Fig. 7f). OSKM-specific H3K27ac peaks are mainly enriched within regions that play a role in neuron development and Wnt and calcium signaling pathways (Supplementary Fig. 7g), while GETM-specific peaks are enriched within regions that involve the regulation of MAPK activity, response to reactive oxygen species (ROS) and metabolic processes (Supplementary Fig. 7h).

As the transcriptome and methylation data, these findings suggest that OSKM initially open (ATAC-seq peaks) and define (H3K4me2 peaks) regions along the genome that have high differentiation potential, but then eliminate their activity by removing the active mark, H3K27ac. In contrast, GETM specifically open and activate regions that are essential for trophoblast function as the process progresses, while closing and methylating regions participating in the embryonic development program.

### Data integration during TSC and pluripotent state induction

**Fig. 7:**
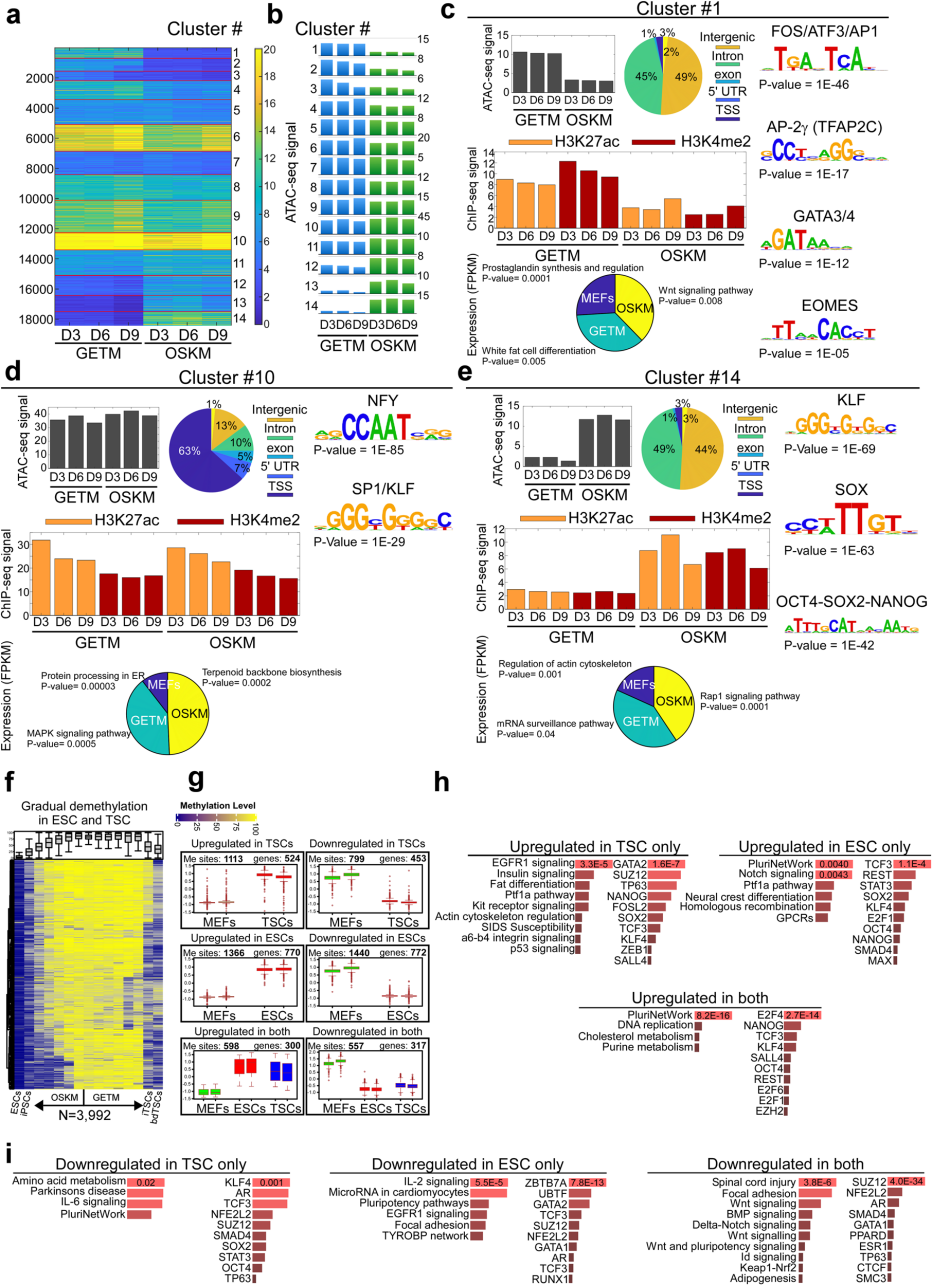
Data integration of DNA methylation, gene expression and chromatin accessibility and activity during reprogramming toward pluripotent and TSC states. **a**–**b** Clustering of 18,420 GETM and OSKM ATAC-seq peaks from days 3, 6, 9 into 14 clusters is shown as a heatmap (**a**) or barplot of mean ATAC-seq signal per cluster (**b**). **c** Cluster #1 is mostly composed of distal (Intergenic and Intronic) GETM-specific peaks, enriched for AP1, Tfap2c, GATA and Eomes motifs, and near GETM-expressed genes. Shown are mean ATAC-seq signals (top left), analysis of their genomic annotations (pie chart, center), enriched transcription factor motifs (right panel), average ChIP-seq signals of H3K27ac and H3K4me2 following GETM and OSKM induction (middle panel), and a pie chart for RNA expression levels and GO term for genes that are associated with each cluster ATAC-seq peaks and exhibit the highest expression levels in MEFs (blue), or GETM (green) or OSKM (yellow, Bottom panel). **d** Same for cluster 10, enriched for highly accessible promoter peaks. **e** Same for cluster 14, with regions that are highly accessible following OSKM, enriched for distal regions with KLF, SOX and OCT4 motifs, and are associated with OSKM expressed genes. **f** A heatmap of differentially methylated blocks with DNA demethylation during the final states of reprogramming to both pluripotent and TSC states. Each row represents one block of DMBs. Boxplots at the top of the heatmap depict the DNA methylation level across bulk samples (two biologically independent replicates per time point/samples, *n* = 3992) during reprogramming toward both pluripotent and TSC states. Boxes indicate 50% (25–75%) and whiskers (5–95%) of all measurements, with black lines depicting the medians. **g** Boxplots (two biologically independent replicates per sample, *n* = 524 (left, top), 770 (left, middle), 300 (left, bottom), 453 (right, top), 772 (right, middle), 317 (right, bottom) of relative expression of differentially expressed genes that are associated with each individual block of DNA methylation. Boxes indicate 50% (25–75%) and whiskers (5–95%) of all measurements, with middle lines depicting the medians. **h**–**i** EnrichR Mouse gene atlas and KEGG pathways analysis of significantly over-represented genes that are either upregulated (**h**) or downregulated (**i**) for each depicted group in (**g**).

We performed data integration analysis to correlate gene expression to chromatin accessibility and activity (Fig. 7a–e, Supplementary Fig. 8a–k) and to methylation (Fig. 7f–i). An initial cluster analysis on 18,420 GETM and OSKM-specific ATAC-seq peaks, yielded 14 clusters (Fig. 7a, b) showing unique patterns of chromatin accessibility, activity, peak distribution and DNA binding motifs in the two reprogramming systems. In each cluster, ATAC-seq peaks were associated to their neighboring genes and their expression compared between GETM induced cells, OSKM induced cells and MEFs (depicted as a pie graph at the bottom of each cluster). Finally, for each group of genes (i.e., highest in GETM, OSKM or MEFs) we performed GO annotation.

Clusters 1–4 are GETM-specific, as in GETM samples they harbor regions with a higher chromatin accessibility and activity compared to OSKM samples (Fig. 7c, Supplementary Fig. 8a–c). While clusters 1–3 are enriched for binding motifs of the GETM reprogramming factors GATA3, TFAP2C and EOMES as well as TBX6 and FOS/ATF3/AP1, cluster 4 is enriched for binding motifs for AP1 and for the master TSC regulator, TEAD^53^. Moreover, while clusters 1-3 contain mostly intronic and intergenic ATAC-seq peaks, cluster 4 encompasses a large fraction of transcription start site (TSS) ATAC-seq peaks. GO annotation analysis revealed that clusters 1–3 include genes related to fat differentiation, p38-MAPK pathway and Glycogen metabolism, while cluster 4 contains genes involved in apoptosis.

Indeed, lipid droplets formation and glycogen storage are crucial for proper trophoblast function and p38-MAPK pathway controls the invasiveness capability of trophoblastic cells^54–56^. Genes that are highest in OSKM-induced cells in clusters 1–3 are related to Wnt signaling, IGF-1 pathway and cardiomyocyte differentiation, while in cluster 4 to BMP signaling pathway. As Wnt and IGF-1 pathways are implicated in pluripotency^57^, it is reasonable to assume that these regions are acting as repressive regions as GETM expressed these genes to a lesser extent than OSKM. Similarly, genes that are expressed to the highest level in the parental MEFs in clusters 1–3 are connected to prostaglandin synthesis, integrin binding and Tgf-β signaling and in cluster 4 to focal adhesion, all implicated in fibroblastic identity maintenance^58,59^ and negatively regulated by GETM factors.

Clusters 5–11 harbor *genomic loc*i with chromatin accessibility and activity which are similar in GETM- and OSKM-induced cells (Fig. 7d, Supplementary Fig. 8d–i). Clusters 6,8,9,10 are highly enriched with strong peaks around the TSS and as such, contain binding sites for transcription factors implicated in transcription initiation, such as NFY, SP1/KLF, ETS, E2A and Oct2^60–62^. Intriguingly, genes of these clusters reaching the highest upregulation in GETM reprogramming are implicated in mRNA processing, while genes exhibiting highest expression in OSKM reprogramming involve mitotic cell cycle regulation, estrogen signaling and insulin signaling, processes that characterize both states. In MEFs, the most highly expressed genes participate in protein processing in ER, EGFR1 signaling, cyclic nucleotide catabolism and cell junction assembly. In contrast, clusters 5,7,11, which are also shared between OSKM and GETM reprogramming but are enriched with smaller peaks around the TSS as in intergenic and intronic regions, contain binding sites, for transcription factors driving the fibroblastic identity (ZEB1, TCF12, TBX5 and SOX6) and more significantly, for the insulator gene *Ctcf*. GO analysis likewise revealed that the highest expression in MEFs in these clusters is for gene ontologies of extracellular matrix organization and integrin binding. Similar expression levels in GETM-induced cells are in genes involved in RNA processing, translation and cytokines biosynthesis, while genes that are highest in OSKM-induced cells are enriched for axonal transport, negative regulation on bone remodeling and ubiquitin conjugation binding, suggesting the initial opening of the different lineages.

Clusters 12–14 are OSKM-specific. Open and active peaks in OSKM-induced cells are enriched for OSK reprogramming factor binding sites, OCT, SOX, KLF and NANOG (Fig. 7e, Supplementary Fig. 8j, k). As GETM-specific clusters, the OSKM-specific clusters are also enriched with peaks localized mostly to intronic and intergenic regions, their associated genes with the highest expression level involving Rap1 signaling pathways, positive regulation of the non-canonical Wnt signaling and neuron development, all implicated in neuronal cell activity^63,64^, further explaining why OSKM reprogramming can induce neuronal fate. Genes with the highest expression levels in GETM induced-cells, play a role in ERBB signaling pathway, glycogen metabolism and mRNA surveillance pathway, all active in trophoblast differentiation. Finally, genes associated with these peaks that exhibit the highest levels in MEFs, are implicated in regulation of actin cytoskeleton and of cell migration, important for normal fibroblastic function.

In conclusion, these data describe how GETM and OSKM reprogramming differ in the induction and dynamics of various signaling pathways, metabolomic processes and their ability to erase the somatic identity and induce alternative cell fates during reprogramming. Moreover, it demonstrates that from the onset of the reprogramming process, GETM reprogramming is directed toward the TSC fate and activates processes and pathways that are essential for TSC maintenance and differentiation.

To correlate gene expression to methylation, we focused on one particularly interesting cluster in which demethylation occurs in most regions of both systems only at the final step of the reprogramming process (Fig. 7f). Associating the 3992 tiles of this cluster to their neighboring genes to examine their expression in ESCs and TSCs (Fig. 7g), revealed 525 genes that are upregulated and 453 genes that are downregulated specifically in TSCs when compared to MEFs. In ESCs, 770 genes were specifically upregulated and 772 downregulated, while there were 300 upregulated and 317 downregulated genes in both ESCs and TSCs compared to MEFs.

GO annotation analysis revealed that such TSCs-specific genes play a role in EGFR1 signaling, insulin signaling and fat differentiation, and contain transcription factor binding sites of GATA2, SUZ12 and TP63, all important for trophoblast formation and differentiation. The most significant biological processes of ESCs-specific genes were pluripotency and notch signaling pathways, with transcription factor binding sites that are enriched for pluripotency genes (*Tcf3*, *Rest*, *Stat3*, *Sox2*, *Klf4*, *Nanog* and *Oct4)*. Genes that are upregulated in both ESCs and TSCs are involved in stemness at large, as pluripotency network and DNA replication were identified as the most significant biological processes and ESC lines and placenta as the most significant tissues. In accordance with that, E2F4 and to a lesser extent other E2F family members, having a key role in stem cell proliferation^65^, were found to be the most significantly enriched binding sites in these tiles, in addition to binding motifs of pluripotent factors such as NANOG, TCF3, KLF4, SALL4 and OCT4 (Fig. 7h). Genes downregulated in TSCs, ESCs, or both, were implicated in fibroblastic identity and function (Fig. 7i), their associated tiles being enriched with binding motifs of factors known to induce strong transcriptional repression such KLF4, AR, ZBTB7A, UBTF, SUZ12 and NFE2L2, suggesting how these hypomethylated regions are associated with gene repression.

This analysis allowed us to examine the final stage of demethylation in both GETM and OSKM reprogramming, which shapes the stabilization stage of both cell types. Surprisingly, this stabilization step still involves the erasure of the fibroblastic identity.

### Genomic stability analysis in GETM and OSKM reprogramming

TSCs have a unique methylation landscape that enables activation of many repetitive elements within the trophoblast genome^66^. This property is believed to induce genomic instability^67,68^ and indeed, multiple genomic aberrations are found in both iTSCs and bdTSCs following prolonged culturing^13^.

Therefore, we inquired whether GETM activation induces genomic instability already at the reprogramming onset toward the TSC state. Myc being a known driver of genomic instability^69^, we reprogrammed fibroblasts into iTSCs by GET or GETM and to iPSCs by OSK or OSKM as a control. Cells were collected for copy number variation (CNV) analysis immediately following infection (day 0), and on day 3 and 6 of reprogramming. We also examined ten iPSC clones and two previously characterized, partially reprogrammed cells^28^ as reference. Genomic reads were aligned against the parental MEF genome. As can be seen in Supplementary Fig. 8l, while many CNVs were identified in one of the two partially-reprogrammed iPSC clones and few CNVs in chromosome 1 or 8 in three out of ten fully-reprogrammed iPSC clones, this analysis could not identify a significant amount of CNVs in the initial phase of either OSK/M or GET/M reprogramming (Supplementary Fig. 8l).

As bulk whole-genome sequencing does not allow the detection of CNVs at single-cell resolution, further examination is needed to fully address this question. However, we can confidently conclude that substantial genomic instability is not induced by GETM at the initial phase of reprogramming.

## Discussion

It has been shown that during human OSKM reprogramming to iPSCs, a subpopulation of cells acquires a TSC fate^70,71^. This is in contrast to the mouse system where only differentiated trophoblasts can be emerge during OSKM reprogramming as assessed by gene expression signatures^72^.

Since pluripotency and TE fates arise simultaneously during blastocyst development, we hypothesized that parallel, comparative multi-omics analysis of both reprogramming processes will yield knowledge that cannot be revealed when each reprogramming is analyzed separately.

By reprogramming MEFs to iPSCs by OSKM, and to iTSCs by GETM, we could examine their transcriptome (Bulk RNA-seq and scRNA-seq), methylome (RRBS), chromatin accessibility and activity (ATAC-seq and ChIP-seq for H3K4me2 and H3K27ac) and genomic stability (CNVs) at various time points, to inquire whether the process toward pluripotent and TSC states follows the same dynamics as during early embryogenesis.

Our analysis revealed that cells undergoing reprogramming to pluripotent and TSC states, exhibit unique and specific trajectories from the process onset till the end, suggesting ‘V’-like behavior. Although similar processes such as somatic identity loss, proliferation, MET and metabolic shift occur in the two systems, each mostly uses different sets of genes and regulatory elements to induce its fate. The ‘V’-shaped behavior was observed at all levels, from transcription and chromatin accessibility and activity, to DNA methylation.

We show that each reprogramming process uses different genomic regions and various strategies to silence fibroblastic identity. While the OSKM combination is potent in inducing identity loss by interacting from its onset with key regions that safeguard fibroblastic identity (enriched with ATF/CREB/AP1 sites), GETM open regions enriched with ATF/CREB/AP1 binding sites, counteracting their ability to silence the fibroblastic identity. This is in agreement with the scRNA-seq data that revealed a large fraction of cells with MEF-like identity even at day 12 of GETM reprogramming.

By harnessing single-cell analysis, we demonstrate two unique and distinct populations of induced cells, suggesting that neither of the induced cells harbor a transcriptional profile that is shared during GETM and OSKM reprogramming. Moreover, we could also illuminate previously unknown stages, markers, blockers and facilitators for OSKM and GETM reprogramming.

Moreover, scRNA-seq analysis for stable iTSCs revealed heterogeneous population of cells, whereby only a small fraction of cells is equivalent to the TE compartment of the pre-implantation blastocyst. This may suggest that sorting-based approaches may be used to isolate this unique subpopulation in an attempt to improve the capability of TSCs to contribute to developing placenta and blastoids.

These results demonstrate that the reprogramming process of somatic cells toward pluripotency and TSC state takes separate routes, and that somatic nuclear reprogramming and reprogramming during early embryonic development toward pluripotency and TE state are characterized by different properties and follow diverse paths.

However, we believe that key features that characterize the process of nuclear reprogramming by OSKM and GETM are shared with the reprogramming process that occurs before lineage specification in the early embryo. As such, by comparing OSKM to GETM reprogramming we show that GETM factors induce DNA methylation on key developmental genes and thus shutting off early embryonic development program responsible for the formation of the brain and heart. By inducing chromatin accessibility and activity and by increasing the levels of the active histone mark H3K27ac, GETM activate the trophoblastic program that involves the activation of metabolic processes that participate in transcription and translation, as well as migration and endothelial cell attraction, which are all known properties of trophoblast cells.

Overall, this study describes and illuminates key features that characterize the reprogramming process toward pluripotent and TSC states at all levels of regulation (i.e., DNA methylation, chromatin accessibility and activity, transcriptome and CNVs), and provide a powerful tool to study cellular plasticity and cell fate decision.

## Methods

### Cell culture and primary MEFs production

iPSCs and BYKE ESCs^23^ (C57BL/6;129/Sv) were cultured in mouse embryonic stem cell medium containing 500 ml DMEM supplemented with (15%FBS, 2 mM L-Glutamine, 1% non-essential amino acid, in-house mouse Leukemia inhibitory factor (mLif), 0.1 mM β-mercaptoethanol (Sigma), 1% penicillin-streptomycin and with or without 2i- PD0325901 (1 μM) and CHIR99021 (3 μM) (PeproTech). All TSCs (derived from blastocysts at the Buganim lab) and iTSCs (C57BL/6;129/Sv) were grown in TSC medium containing a combination of 70% MEF conditioned medium and 30% freshly prepared medium, (RPMI supplemented with 20%FBS, 0.1 mM β-mercaptoethanol, 2 mM L-Glutamine, 1% penicillin-streptomycin, 25 ng/ml human recombinant FGF4 (PeproTech) and 1 μg/ml heparin (Sigma-Aldrich) or in a defined TX medium (in the case of the single cell analysis) as previously described^30^. Mouse embryonic fibroblasts (MEFs) were isolated as previously described^73^. Briefly, embryos from OCT4-GFP mice or chimeric embryos, derived from BYKE ESC injection into blastocysts, were isolated at E13.5 and then dissected under the binocular to remove any internal organs and heads. The tissue was chopped by scalpels and incubated 30 min with 1 ml Trypsin-EDTA (0.25%, Gibco) at 37 °C. Next, trypsin activation was neutralized by 10 ml DMEM containing 10% serum and the chopped embryos underwent intensive pipetting until homogeneous mixture of cells was noted. Each embryo was seeded into one 15 cm plate and cultured with DMEM containing 10%FBS, 1% penicillin-streptomycin and 2 mM L-glutamine. The cells were grown till the plate being full. Puromycin (2 µg/ml) was added for selection for BYKE MEFs (the M2rtTA cassette that resides inside the rosa26 locus of the injected cells contains a resistance gene for puromycin), eliminating only the host cells. All cells were maintained in a humidified incubator at 37 °C and 6% CO2.

### Chimeric embryo production

Blastocyst injections were performed using CB6F1 host embryos. After priming with PMSG (PROSPEC, hor-272b) and hCG (EMD Millipore, 000725) hormones and mating with CB6F1 males, embryos were obtained at 3.5dpc (blastocyst stage) for chimera assay. 8–12 ESCs were injected into 3.5dpc blastocysts with a flat tip microinjection pipette with an internal diameter of 16 μm (Origio Inc, Charlottesville, VA, HU-Piezo-16-15) in drop of Evolve^®^ w/HEPES KSOMaa (Zenith, ZEKS-050) medium under mineral oil. Shortly after injection, blastocysts were transferred to 2.5dpc pseudopregnant CD1 females (~20 blastocysts per female). The joint ethics committee (IACUC) of the Hebrew University and Hadassah Medical Center approved the study protocol (IACUC# MD-17-15286-3) for animal welfare. The Hebrew University is an AAALAC international accredited institute.

### Molecular cloning, lentiviral infection, and reprogramming

The open reading frame of the examined genes (i.e., Ctcf^28^, Cdx2^13^, Atf3^74^, Tead2, Fosl2, Pdx1, Nrf2, Usf1, Usf2, NE-F2, Fos, MafK, Tcf15, E2f4, Nr1h4 and Rfx2) was cloned into pMINI vector (NEB) and then restricted with EcoRI or MfeI and transferred into FUW-TetO expression vector. Lentiviruses were generated by transfecting vector DNA, (hGETM 3:3:3:1) or STEMCCA cassette for hOSKM, with a mix of lentiviral packaging vectors (7.5 µg psPAX2 and 2.5 µg pGDM.2) into 293 T cells, the viruses were collected at 48, 60, and 72 h after transfection, the medium containing the viruses was supplemented with 8 µg/ml of polybrene (Sigma) and filtered by 0.45 µm filter, the viruses were then added to MEFs (passage 0) that were seeded at 70% confluency 2 days prior to the first infection. Six hours following the third infection, medium was changed into DMEM containing 10%FBS. Eighteen hours later, medium was changed into reprogramming medium; ESC reprogramming medium (DMEM supplemented with 10%FBS, 0.1 mM β-mercaptoethanol, 2 mM L-glutamine, 1%non-essential amino acids, in-house mouse Leukemia inhibitory factor (mLif), and 2μg/ml doxycycline) or TSC reprograming medium (RPMI supplemented with 20%FBS, 0.1 mM β-mercaptoethanol, 2 mM L-glutamine, in house mouse recombinant FGF4 (equivalent to 25 ng/ml), 1 μg/ml heparin (Sigma-Aldrich), and 2 μg/ml doxycycline). The two reprogramming mediums were changed every other day. For iPSC reprogramming, MEFs were exposed to doxycycline for 15 days, followed by 5 days of dox withdrawal in ESC culturing medium. For iTSC reprogramming, MEFs were exposed to doxycycline for 20 days, followed by 10 days of dox removal in TSC culturing medium. iTSCs colonies were then isolated, trypsinized, and plated in a well in a six-well plate on feeder cells and passaged until stable colonies emerged.

### Quantitative PCR

Total RNA was isolated using the Macherey-Nagel kit (Ornat). 1000 ng of total RNA was reverse transcribed using iScript cDNA Synthesis kit (Bio-Rad). Quantitative PCR analysis was performed in duplicates using 1/100 of the reverse transcription reaction in a StepOnePlus (Applied Biosystems) with SYBR green Fast qPCR Mix (Applied Biosystems). Specific primers flanking an intron were designed for the different genes (for primer sequences see Table 1). All quantitative real-time PCR experiments were repeated at least three times, and the results were normalized to the expression of *Gapdh* and presented as a mean ± standard deviation of two duplicate runs from a typical experiment.

**Table 1:** List of primers.

Gene	Primers
Mafk^cDNA^	F- 5′ CCGGGTTATGACGACTAATCC 3′
	R- 5′ GAGCCTGGGATAGGCATGAG 3′
Fosl2^cDNA^	F- 5′ AAACCACCCTGTTTCCTCTC 3′
	R- 5′ ACCAGTGTCTCACCACTAAG 3′
Nrf2^cDNA^	F- 5′ CAGTTGCCACCCAGGATGTC 3′
	R- 5′ GGGTTTACTCGTCAGTAGTG 3′
Pdx1^cDNA^	F- 5′ ATGAACAGTGAGGAGCAGT 3′
	R- 5′ TCACCGGGGTTCCTGCGGT 3′
Nfe2^cDNA^	F- 5′ GGCTTTCAGCTGGCACAGTAG 3′
	R- 5′ GGCTTTGAGGGAGTCTCTAGC 3′
Usf1^cDNA^	^94^
Usf2^cDNA^	^94^
Atf3^cDNA^	^95^
Rfx2^cDNA^	F- 5′ TCCGGAAGCACGTGGAAGAC 3’
	R- 5′ GAGAGTCCAGGTCCCTAGAG 3'
hTEAD2^cDNA^	F- 5′ GGAATCGGGATCCTGCTTGG 3’
	R- 5′ CCGGTTCCTTTCTAAGAGGAG 3'
hFOS^cDNA^	F- 5′ AGCTCCCACCAGTGTCTACC 3’
	R- 5′ TTGCCTTCTCTGACTGCTCAC 3'
hGATA3^cDNA^	F- 5′ ATGGAGGTGACGGCGGACCAG 3’
	R- 5′ CTAACCCATGGCGGTGACCATGC 3'
hTFAP2C^cDNA^	F- 5′ ATGTTGTGGAAAATAACCGATA 3′
	R- 5′ TTATTTCCTGTGTTTCTCCATTT 3′
hEOMES^cDNA^	F- 5′ ATGCAGTTAGGGGAGCAGCTCTTG 3′
	R- 5′ TTAGGGAGTTGTGTAAAAAGC 3′
hE2F4^cDNA^	The open reading frame was synthesized by TWIST (Cat#: tSHPs0623B597503QG)
hTCF15^cDNA^	The open reading frame was synthesized by TWIST (Cat#: tSHPs0623B597501QG)
hNR1H4^cDNA^	The open reading frame was synthesized by TWIST (Cat#: tSHPs0623B597502QG)
Gapdh	F‐ 5′ ACCTGCCAAGTATGATGACATCA 3′
	R‐ 5′ CCCTCAGATGCCTGCTTCAC 3′
Nccrp1	F- 5′ AGCTCACCCAACCCAGAAG 3′
	R- 5′ TCCACGGAAATTACCCAGCT 3′
Ehf	F- 5′ AGTCTGCAGGAGTTCACGAG 3′
	R- 5′ TTGTGTGCGGACTGGAAAAG 3′
Krt17	F- 5′ CAAGATCCTTGTGGCCACC 3′
	R- 5′ AGCCTGCTCTGTCTCAAACT 3′
Slc38a3	F- 5′ CAATACGGGCATCATCCTTT 3′
	R- 5′ AGACTTGAGGAGCAGGTGGA 3′
Plac1	F- 5′ GGGAGGCACTGTCTTAGTCG 3′
	R- 5′ AACGGTGACCATGAACCAAT 3′
Prl8a9	F- 5′ GACAGCTGGAACCCTTCGTA 3′
	R- 5′ CAGCTCTGGCAACAGTCTCA 3′
FUW-tetO vector^transgenic^	F- 5′ CGCCTGGAGACGCCATCCACGCTG 3′
Ctcf^transgenic^	R- 5′ GACTCCTCCACAATGGCTTC 3′
hTEAD2^transgenic^	R- 5′ CTCCTCACTGCCTTCCTCAC 3′
Atf3^transgenic^	R- 5′ GTGAGAGGCAGGGGACAAT 3′
Fosl2^transgenic^	R- 5′ GGACGAGGTGTCAAAGTTCC 3′
Pdx1^transgenic^	R- 5′ CACGGGTCCTTGTAGAGCTG 3′
Usf1^transgenic^	R- 5′ AAAGTGGCAGCTGACTGGAT 3′
Usf2^transgenic^	R- 5′ GGAAGCGGGATCCAGACC 3′
Nfe2^transgenic^	R- 5′ CCAACAGGCAGCTGTGATAA 3′
hFOS^transgenic^	R- 5′ GTCTGCGGGTGAGTGGTAGT 3′
Mafk^transgenic^	R- 5′ TGACATGGACACCAGCTCAT 3′
KLf4^transgenic^	R- 5′ ACGCAGTGTCTTCTCCCTTC 3′
Nrf2^transgenic^	R- 5′ CTCATAGTCCTTCTGTCGCTGA 3′
Rfx2^transgenic^	R- 5′ CACTGACGCTGGCGAATCTG 3′
hE2F4^transgenic^	R- 5′ CGGCGAGTTTCAGATCCAG 3′
hTCF15^transgenic^	R- 5′ TGGTCTCAGGAGTGCAAATG 3′
hNR1H4^transgenic^	R- 5′ TTCATTTTTGATCCCATC 3′

### Flow cytometry

Cells were trypsinized, washed with PBS×1 and filtered through mesh paper. Samples were analyzed by a Beckman Coulter (Gallios) flow cytometer using the Kaluza Software (V 1.0.14029.14028).

### Immunofluorescence

Cells were fixed in 4% paraformaldehyde in PBS for 20 min. The cells were rinsed three times with PBS and blocked for 1 h with PBS containing 0.1% Triton X-100 and 5% FBS. The cells were incubated O/N in PBS containing 0.1% Triton X-100 and 1% FBS with primary antibodies in 4 °C. The antibodies that were used are: anti-CDX2 (Biogenex, CDX2-88, 1:000) and anti-TACSTD2 (TROP2, Abcam, ab214488, 1:500). The next day, the cells were washed three times and incubated for 1 h with relevant secondary antibodies (goat Anti-Mouse IgG (Alexa Fluor 488, Ab150113, 1:500) and Goat Anti-Rabbit IgG (Alexa Fluor 594, Ab150080, 1:500) in PBS containing 0.1% Triton X-100 and 1% FBS. DAPI (1:1000 dilution) was added 10 min before the end of the incubation and stained cells were inspected under Nikon Eclipse Ti fluorescent microscope.

### Quantification and statistical analysis

Unpaired Student’s *t* test was used for experiments comparing differences between two groups. Statistical significance differences were considered when *p* value ≤ 0.05. All experiments were repeated at least three times. For quantitative PCR experiments the results were normalized to the expression of the housekeeping control gene, *Gapdh* from two duplicate runs from a typical experiment. Unless indicated otherwise a representative experiment is shown for each Figure.

### RNA libraries and sequencing

Total RNA was isolated using the Qiagen RNeasy kit. mRNA libraries were prepared using the SENSE mRNA-seq library prep kit V2 (Lexogen), and pooled libraries were sequenced on an Illumina NextSeq 500 platform to generate 75 bp single-end reads.

### Reduced representation bisulfite sequencing (RRBS)

RRBS assay was performed as previously described^75^. Briefly, 20 ng of genomic DNA were digested with Msp1 restriction enzyme (NEB, R0106L), DNA fragments were end-repaired and A-Tailed using Klenow fragment (3′−5- exo-) (NEB, M0212L). The DNA fragments were then ligated to illumina adaptors (Illumina, PE-940-2001) using T4 ligase (NEB, M0202M) and then size selected using AMPure XP beads (Beckman Coulter Genomics, A63881). The samples were then subjected to two consecutive bisulfite conversions using EpiTect Bisulfite Kit (QIAGEN, 59104) and PCR using PfuTurbo Cx hotstart DNA polymerase (Agilent Technologies, 600412). The RRBS libraries were sequenced by Illumina HiSeq 2000 platform.

### Single-cell RNA-seq

Induced cells at day 3, 6 or 12 and iTSCs were prepared as instructed in the 10× Genomics cell preparation guidelines. Briefly, cells were trypsinized and centrifuged at 1000 RPM for 3 min, then were washed twice with PBS×1 containing 0.04% BSA and cleaned from cell debris and large clumps by filtering throw mesh paper. Next, resuspended cells were subjected to dead cell removal kit (MACS, 130-090-101) to remove any non-viable cells. Cell viability were estimated using trypan blue staining. Cells were then resuspended in PBSx1 with 0.04% BSA at the concentration of 1000 cells/µl and 4000 cells from each condition were subjected to 10× Genomics. Single-cell RNA libraries were prepared using Chromium Single Cell 3′ Library Kit v2 (10× genomics, 120234) and the generated libraries were sequenced using Illumina NextSeq 500 platform.

### Chromatin immunoprecipitation (ChIP)

Chromatin immunoprecipitation (ChIP) assay was performed as previously described^76^. Briefly, cells were fixed for 10 min at RT with a final concentration of 0.8% formaldehyde. Formaldehyde was quenched with glycine for a final concentration of 125 mM. The cells were then lysed with lysis buffer (100 mM Tris-HCl, 300 mM NaCl, 2% Triton^®^ X-100, 0.2%v sodium deoxycholate, 10 mM Cacl2) supplemented with EDTA free protease inhibitor (Roche- 11873580001) for 20 min in Ice and the chromatin was digested by MNase (micrococcal nuclease- Thermo Scientific^™^− 88216) for 20 min at 37 °C. MNase was inactivated by 20 mM EGTA. The fragmented chromatin was incubated with pre-bounded Dynabeads (A and G mix - Invitrogen 10004D/ 10002D) using H3K27ac antibody (Abcam, ab4729, 2 μg/reaction) and H3K4me2 antibody (Millipore, 07-030, 2μg/reaction). Samples were then washed twice with RIPA buffer, twice with RIPA high salt buffer (NaCl 360 mM), twice with LiCl wash buffer (10 mM Tris-Hcl, 250 mM LiCl, 0.5% DOC, 1 mM EDTA, 0.5% IGEPAL) and twice with 10 mM Tris-HCl pH = 8. DNA was purified by incubating the samples with RNAse A (Thermo Scientific^™^ EN0531) for 30 min at 37 °C followed by a 2 h incubation with Proteinase K (Invitrogen^™^ 25530049). DNA was eluted by adding 2× concentrated elution buffer (10 mM Tris-HCl, 300 mM NaCl, 1% SDS, 2 mM EDTA) and then reverse crosslinked overnight at 65 °C. Finally, DNA was extracted using AMPure XP beads (Beckman Coulter Genomics, A63881). Chip sample libraries were prepared according to Illumina Genomic DNA protocol as described^77^. Briefly, 10 ng of the fragments were end-repaired by T4 Polynucleotide Kinase and T4 DNA Polymerase (NEB, M0201S, M0203S), A-tailed (NEB, M0212S), and ligated to illumina adaptors (Illumina, PE-940-2001) using T4 ligase (NEB, M0202M). The libraries were amplified for eight cycles using NEBNext^®^ Ultra^™^ II Q5^®^ Master Mix (NEB, M0544), size selected using AMPure XP beads (Beckman Coulter Genomics, A63881), and sequenced on an Illumina NextSeq 500 platform.

### ATAC libraries and sequencing

ATAC-seq library preparation was performed as previously described^78^. Briefly, cells were trypsinized and 50,000 cells were counted and incubated in lysis buffer to isolate nuclei. Nuclei were then resuspended in transposase reaction mix for 30 min at 37 °C (Illumina, Fc-121-1030). The samples were purified using Qiagen MiniElute kit (QIAGEN, 28204), Transposed fragments were directly PCR amplified and sequenced on an Illumina NextSeq 500 platform to generate 2 × 36 bp paired-end reads.

### Data processing

#### Read quality control

Read quality for ChIP-seq, ATAC-seq, RNA-seq, scRNA-seq, and RRBS was examined using FastQC (V 0.11.8, [https://www.bioinformatics.babraham.ac.uk/projects/fastqc/]). Quality of ChIP-seq and ATAC-seq peaks was assessed using ChIPQC (V 1.24.1)^79^. Quality of the RNA-seq data alignment was assessed using RSeQC (V 4.0.0)^80^. Bisulfite conversion rates for all RRBS samples were estimated using MethylDackel (V 0.5.1) [https://github.com/dpryan79/MethylDackel]. Single-cell RNA libraries were prepared using two different kits for the two batches. The first batch that includes day 6 and day 12 was prepared using Chromium Single Cell 3′ Library Kit v2 and second batch that includes day 3 and iTSCs was prepared using Chromium Single Cell 3′ Library Kit v3. In order to remove the batch effect, we combined the two batches together and utilized Seurat’s batch correction pipeline using SelectIntegrationFeatures, FindIntegrationAnchors, and IntegrateData.

#### Bulk RNA-seq

Low quality bases and sequencing adaptors of 36 raw fastq files RNA-seq containing single-end 61 bp-long reads were trimmed using Trim Galore (V 0.6.0, [https://github.com/FelixKrueger/TrimGalore]) and then mapped to the mm9 reference genome using HISAT2 (V 2.1.0^81^,) with default parameters. Read counting was performed using featureCounts (V 1.6.2^82^) with (Mus_musculus.NCBI37.gtf annotation). Differential gene expression analysis was performed using DESeq2_1.26.0 package^82^. Unsupervised hierarchical clustering was performed for 10,000 most variable genes among ESCs, bdTSCs, fibroblasts and cells during reprogramming. R package dynamicTreeCut^83^ was used to perform adaptive branch pruning detecting 27 prominent clusters. R packages EnrichR (V 2.1^84^,) and ClusterProfiler (V 3.14.3^85^,) were used to query Biological processes, Mouse gene atlas and KEGG pathways analysis of significantly over-represented genes for each cluster. A second aligner TopHat [4] (V 2.0.6^86^,) was used to map reads to mm9 reference genome. Mapped reads were then processed using cufflinks [4] (V 2.0.2^87^), and gene expression levels (FPKM) were calculated for each replicate. Differential gene expression analysis was performed using DESeq2 package (V 1.26).

#### 10× Single-cell data

scRNA-seq libraries were generated from each time point using the 10× Genomics. The cellranger-6.1.1 [https://github.com/10XGenomics/cellranger] was used for mapping of the 10× scRNA-seq data. Read1 data of pooled cells were split into single-cell data using the barcode sequences contained in the first 16 bps. The next 10 bps were recorded as unique molecular identifiers (UMIs). Read2 with 75 bp were aligned to the mm10 reference genome. We used Seurat (V 4.0.5^88^) to pre-processing the data and perform clustering. The function ‘FindAllMarkers’ to identify the marker genes for each of the clusters in the UMAP representation. For day 3 OSKM and GETM reprogramming and iTSCs, 20,000 cells were profiled. Around 7000 cells from each reprogramming system were pooled with 3000 cells of Sox2-GFP stable iTSCs cultured on feeder cells under TSC defined medium, TX^30^. Initial clustering for all the dataset was done to explore and determine quality control cutoffs. Clusters that had an average UMIs ≤10,000 or an average mitochondrion UMIs >10% were excluded. For Day 6 OSKM and GETM reprogramming, we excluded clusters that had an average UMIs <8000 or average mitochondrion UMIs >10%. For Day 12 OSKM and GETM reprogramming, we excluded clusters that had an average UMIs <9000 or average mitochondrion UMIs >10%. R package DoubletFinder (V2.0.3), [https://github.com/chris-mcginnis-ucsf/DoubletFinder] was used to identify and exclude potential doublets. Following quality control (i.e. removal of duplets, lowly expressed cells and mitochondrial RNA-enriched cells) we analyzed a sum of 26,839 cells for the 7 conditions: OSKM: (D3 :4952, D6: 3181, D12: 2835 cells), GETM (D3 :4756, D6: 4625, D12: 3097 cells) and iTSCs: (3393 cells).

#### DNA methylation

Low quality bases and sequencing adaptors of 45 raw fastq files were trimmed using Trim galore (V 0.6.0, [https://github.com/FelixKrueger/TrimGalore]) and then mapped to the mm9 reference genome using Bsmap (V 2.90^88^) with flags -S 10 -R -p 8 -D C-CGG. Bam files belonging to same reprogramming system and day were merged to ensure maximum overlap between all samples. Methylation beta values were extracted from the BAM files using wgbs_tools [https://github.com/nloyfer/wgbs_tools]. Methylation markers were identified using in-house developed script find_markers.py to generate Bed files with *p* value ≤ 0.05 between different conditions summarized in different groups. 130,000 blocks were identified with significant methylation alteration that occurs during reprogramming in both OSKM and GETM reprogramming. In order to minimize noise and extract significant trends, we used the K-means algorithm to classify ~130,000 blocks that are shared amongst all samples during reprogramming to a TSC or pluripotent states and obtained 100 clusters. A new table was constructed by averaging DNA methylation levels per sample per cluster and then projected the processed data onto the first two principal components. Clusters loading plot showed significant clusters contributed to the first two principal components and clusters that are near to each other showed similar trends of methylation allowing us to extract 15 different trends shown as heatmaps in Fig. 4a and Fig. S4a. Genomic regions associated with all blocks belonging to each of the 15 clusters were annotated using GREAT (V 4.0.4^32^) and were summarized in Supplementary Data file 3.

#### ATAC-seq

Fastq files were mapped to the mm9 reference using bwa (V 0.7.17-r1188, [https://arxiv.org/abs/1303.3997]). The mapped reads were converted to BAM format and filtered by mapping quality (MAPQ) of ≥10, retaining only properly aligned pairs (samtools -F 1796 flag). The BAM files were then sorted and indexed using samtools (V 1.9^89^).

Bigwig coverage tracks were generated using deepTools bamCoverage (V 3.4.1^90^) with the following flags: “--normalizeUsing RPGC -bs 50 -e 500 --effectiveGenomeSize 2150570000”. Coverage peaks were called using MACS (V, 2.1.2^91^) with flags “-g mm --slocal = 2000 --llocal = 20000 --nomodel --extsize = 300 -f BAMPE”. Peaks of multiple replicates were retained only if identified by 30% of the replicates, or more.

#### ChIP-seq

Fastq, BAM and bigwig files were processed in a similar way to the ATAC-seq files.

#### Annotation of genomic regions

Peaks from each experiment were divided into subsets, including peaks that appear in both OSKM and GETM (3, 6, or 9 days after induction) but not in MEFs, peaks from GETM (days 3, 6, 9) not identifiable in MEFs, OSKM peaks (days 3, 6, 9) not identifiable in MEFs, and disjoint sets of cell-type specific peaks (e.g., GETM day 3 peaks not found in MEFs or in OSKM day 3, etc.). We also analyzed peaks from ESCs, TSCs or MEFs.

Genomic regions from each group of peaks were then annotated using annotatePeaks.pl (HOMER suite, [http://homer.ucsd.edu/homer/ngs/annotation.html], UCSC mm9 genome version) as Promoter, TTSs, 5′ and 3′ UTRs, or as Exonic, Intronic, or Intergenic regions.

#### Motif analysis

ATAC-seq peaks were called in each replicate separately, and overlapping peaks from replicates were then merged. Peaks overlapping MEF peaks (top 50 K) were then removed. Finally, the center 250 bp of each peak was considered for further analysis (peaks shorter than 250 bp were removed).

We further divided the peaks of each time point into disjoint groups, including peaks identified in both GETM and OSKM ATAC-seq (e.g. GETM&OSKM D 3), GETM-only peaks (e.g. GETM\OSKM D 3) or OSKM-only peaks (e.g. OSKM\GETM D 3).

Finally, we used “findMotifsGenome.pl -nomotif” (HOMER suite) to identify occurrences of known motifs within those sequences. A similar approach was applied to H3K27ac and H3K4me2 ChIP-seq peaks.

#### CNV analysis

Read alignment was done with BWA mem 0.7.15^92^ to the mouse reference genome mm9 including PhiX174. Copy number analysis was performed by cnvkit 0.9.6^93^ using control and control-GFP-IPSCs as reference.

### Reporting summary

Further information on research design is available in the Nature Research Reporting Summary linked to this article.

## Supplementary information

Supplementary Information

Description of Additional Supplementary Files

Supplementary Data 1

Supplementary Data 2

Supplementary Data 3

Reporting Summary

## Data Availability

ATAC-seq, ChIP-seq, RRBS, scRNA-seq, bulk RNA-seq and CNV has been deposited to the Gene Expression Omnibus database (GEO, Accession number GSE171127 [GSE171127). Additional data from Benchetrit et al. are available at GEO, GSE98124. All analyses used UCSC mm9 mouse reference genome [http://genome.ucsc.edu/cgi-bin/hgGateway?db=mm9], except for the 10× single-cell RNA-seq data, which used mm10 [http://genome.ucsc.edu/cgi-bin/hgGateway?db=mm10]. The figures that are associated with the raw data files are: Figs. 2a–m, 3a–i, 4a–e, 5a–j, 6a, b, 7a–i and supplementary Figs: 1e–j, 2a–g, 3a, b, e, f, 4a–g, 5a–c, 6a–h, 7a–h, 8a–i. Remaining data are provided within the Article, Supplementary Information. Source data are provided with this paper.

## Source data

Source Data

## Code availability

DNA methylation data were analyzed using wgbstools, a computational suite for DNA methylation sequencing data representation, visualization, and analysis [https://github.com/nloyfer/wgbs_tools].
