# Supplementary material for: Comparative parallel multi-omics analysis during the induction of pluripotent and trophectoderm states
Source: Nat Commun. 2022 Jun 17;13:3475. doi: 10.1038/s41467-022-31131-8 (PMC9205865; doi:10.1038/s41467-022-31131-8)
Supplement: Supplementary file 1 — Supplementary Information [file 41467_2022_31131_MOESM1_ESM.docx]

**SUPPLEMENTARY DATA**

**
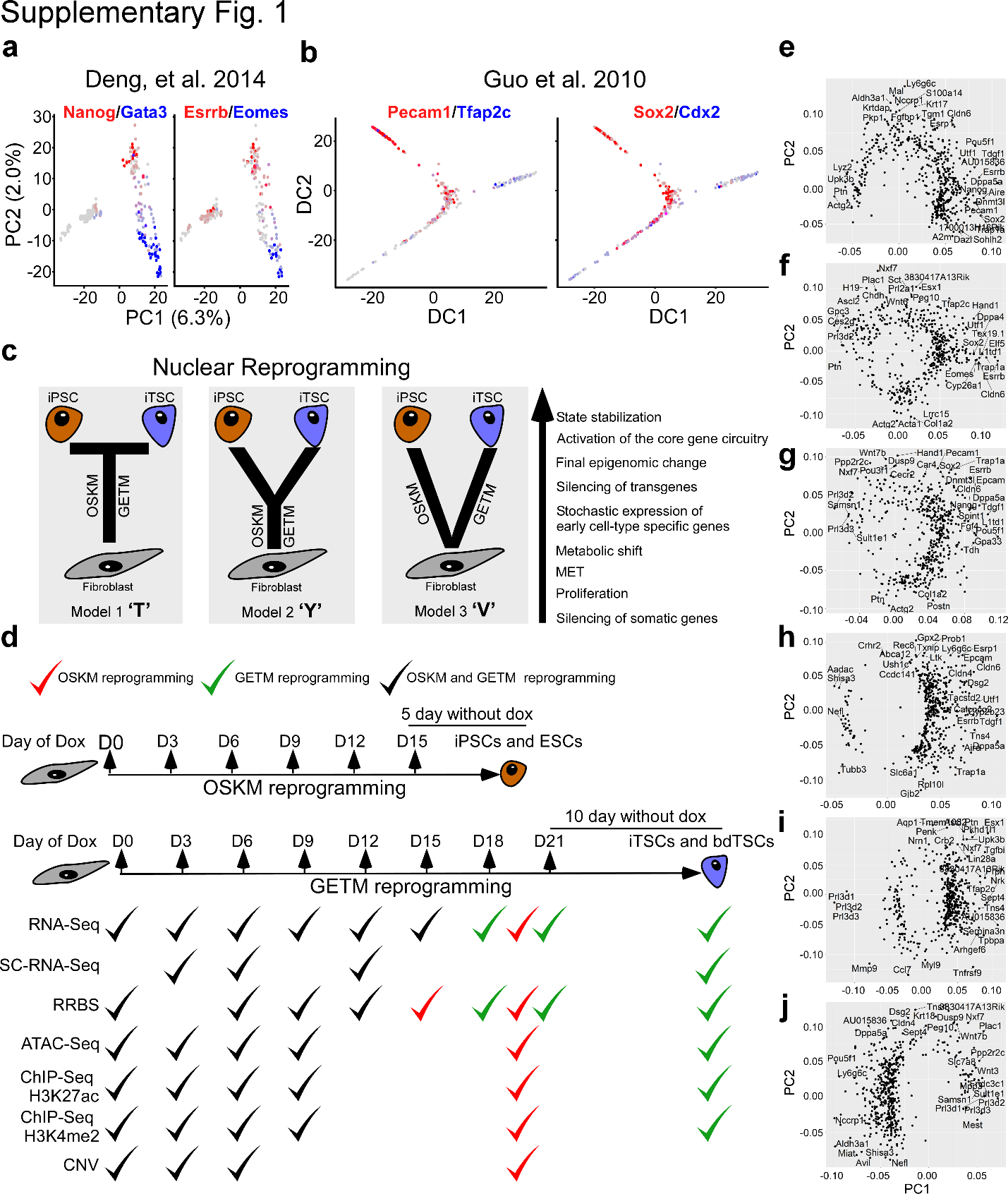
**

**Supplementary Fig. 1. Embryonic development trajectory and hypothetical nuclear reprogramming progression models using comparative multi-omics analysis. (a)** Overplayed co-expression of Nanog/Gata3 and Esrrb/Eomes among individual cells extracted from^5^. Red color indicates cells that are enriched with key pluripotency genes while blue color indicates cells with enrichment of key TE genes. **(b)** Overplayed co-expression of Pecam1/Tfap2c and Sox2/Cdx2 among individual cells extracted from^7^. Red color indicates cells that are enriched with key pluripotency genes while blue color indicates cells with enrichment of key TE genes. **(c)** Schematic illustration of three possible models, ‘T’, ‘Y’, ‘V’, explaining the reprogramming progression of fibroblasts toward either iPSCs by OSKM or iTSCs by GETM. **(d)** Schematic representation of the reprogramming process of fibroblasts to iPSCs (top, red) and iTSCs (bottom, green) and the various high throughput experiments and time points that were analyzed. Black ‘V’ represents a time point that was taken for both GETM and OSKM reprogramming while Green ‘V’ represents GETM-only time point and red ‘V’ represents OSKM-only time point. **(e-g)** PCA loading plots showing the contribution of individual genes out of top 500 most differentially expressed genes to the first and second PCA components; distance from the origin along each axis corresponds to strength of contribution to that component. Shown are PCA loading plots for the reprogramming process to either iPSCs (e), iTSCs (f) or both (g) as assessed by gene expression profiles of bulk RNA-seq data projected onto the first two principal components. 32 highest ranked genes are marked in each plot. **(h-j)** same as in (e-g) but here only induced cells (cells on dox) are plotted.

**
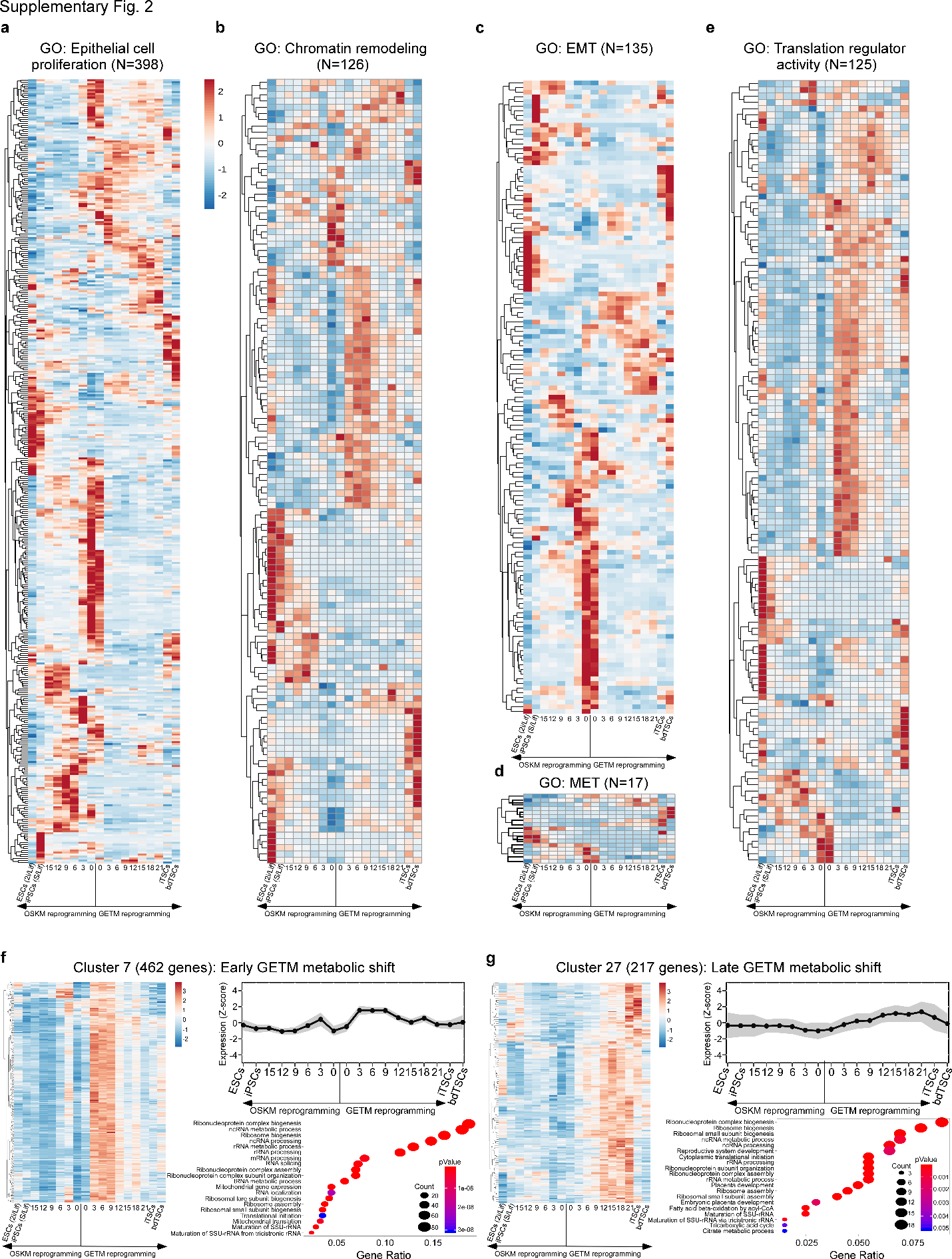
**

**Supplementary Fig. 2. GETM and OSKM reprogramming factors mostly exhibit mutually exclusive transcriptional profiles during reprogramming.** **(a-e)** Heatmaps showing the expression levels of genes involved in early and general reprogramming processes such as epithelial cell proliferation (a), chromatin remodeling (b), EMT (c), MET (d) and translation regulator activity (e), during the conversion of fibroblasts into iPSCs by OSKM and into iTSCs by GETM. **(f)** Heatmap, expression pattern plot and GO terms of 462 genes of cluster #7 as detected by bulk RNA-seq during reprogramming toward iPSCs and iTSCs. **(g)** Heatmap, expression pattern plot and GO terms of 217 genes of cluster #27 as detected by bulk RNA-seq during reprogramming toward iPSCs and iTSCs.

**
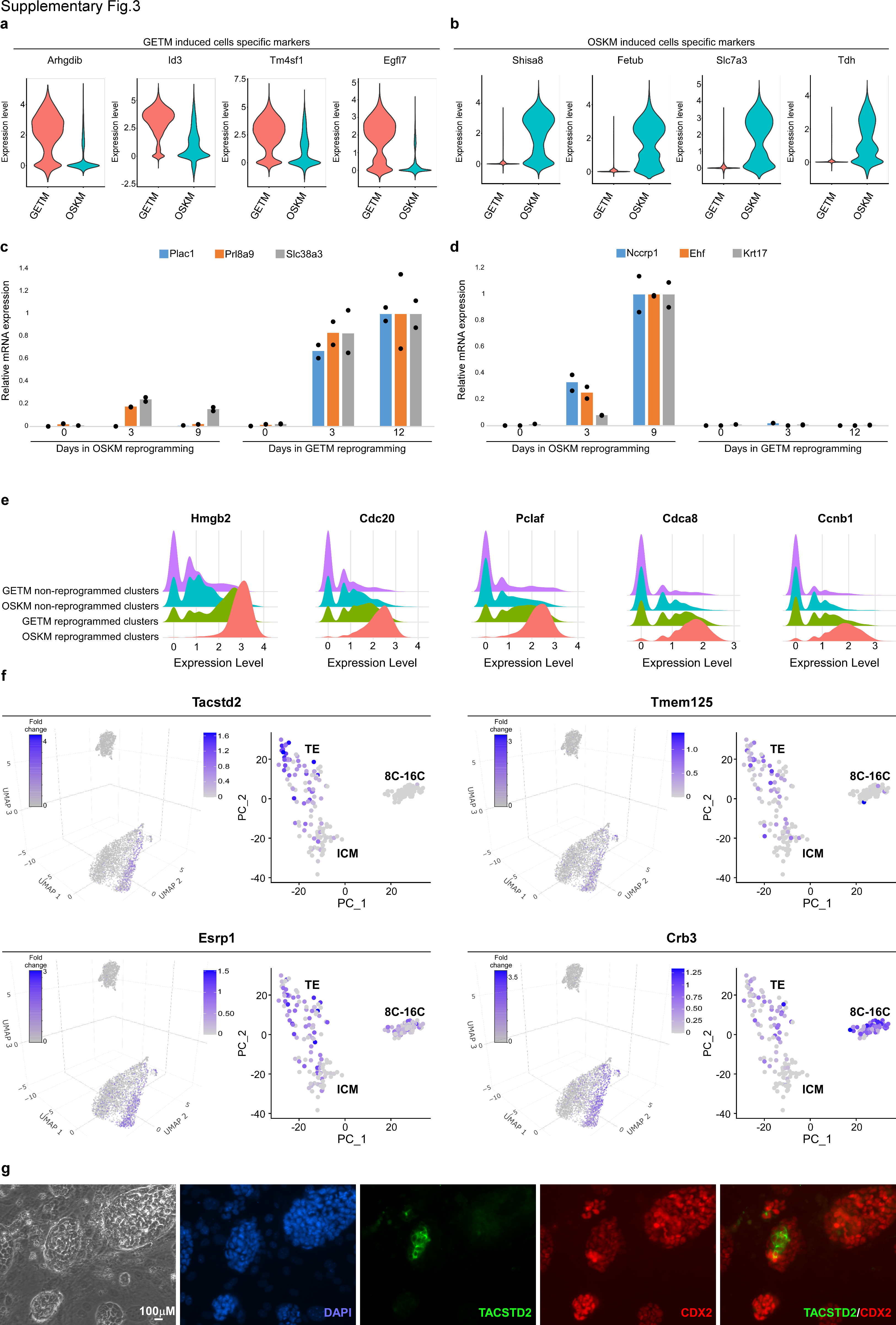
**

**Supplementary Fig. 3. Single-cell RNA-seq analysis on OSKM and GETM reprogrammable cells and iTSCs identifies reprogramming-specific markers and cell heterogeneity. (a, b)** Violin plots summarizing single-cell expression level of GETM-specific markers (a) and OSKM-specific markers (b) that significantly distinguish between the two reprogramming systems. **(c, d)** qPCR analysis of GETM reprogramming-specific genes (c) and OSKM reprogramming-specific genes (d). The highest sample for each gene was set to 1. Results were normalized to the *Gapdh* gene and are shown as fold change using two replicates (n=2) in a typical experiment out of 3 runs. **(e)** Redge plots showing the expression distributions of key proliferation genes where each community represents potentially reprogrammed or non-reprogrammed cells for each reprogramming system. **(f)** Expression level of selected cluster-specific markers showing prevalent expression at the single-cell level for genes shared with the embryo TE state^5^. The expression level of the specified markers is visualized by UMAPs for the various clusters (left) and PCAs (right) for the indicated stages in embryogenesis and quantified by a range of intensities of a purple color. **(g)** Representative bright field and immunostaining images for CDX2 (red), TACSTD2 (green), and DAPI (blue) in bdTSCs grown on feeder cells and defined TX medium (n=2). Source data are provided as a Source Data file.

**
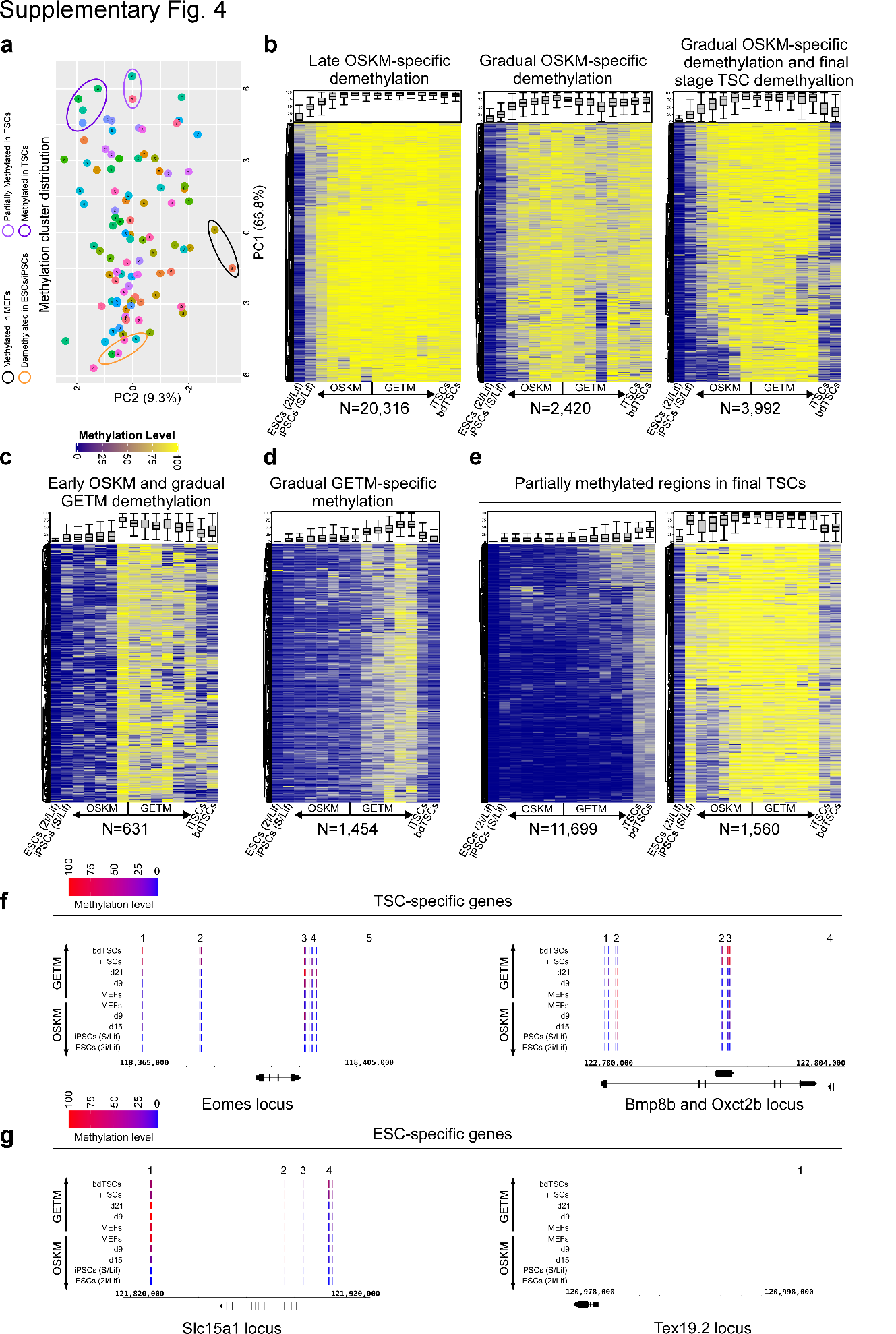
 Supplementary Fig. 4. Methylation dynamics during OSKM and GETM reprogramming**

**(a)** PCA plot projected by the first two principal components showing the average DNA methylation per sample for 100 clusters obtained from a total of 130,000 methylation blocks across all samples. Each color represents a single cluster out of a total of 100 clusters obtained by k-means clustering. Each cluster was then summarized by a single tuple that explains a global methylation average of a particular methylation trend during reprogramming. Clusters that are near to each other show similar trend of methylation. **(b-e)** Heatmaps showing the dynamics of DNA methylation alterations and specific cluster patterns across bulk samples during reprogramming towards both pluripotent and TSC states. Boxplots at the top of each heatmap depict the DNA methylation level across the indicated bulk samples (for each sample and time point two biologically independent replicates were analyzed, (b) n= from left to right: 20,316, 2,420, 3,992, (c) n= 361, (d) n= 1,454, (e) n= 11,669 (left), 1,560 (right)) during reprogramming towards both pluripotent and TSC states. Boxes indicate 50% (25–75%) and whiskers (5–95%) of all measurements, with black lines depicting the medians. **(f)** Genome browser snapshot showing RRBS-captured CpG sites (short blue, purple or red lines) of the indicated samples in *TSC-specific loci* (i.e*., Eomes*, *Bmp8b* and *Oxct2b*). Scale bar indicates methylation levels ranging from no methylation (blue), intermediate methylation (purple) to maximum methylation (red). **(g)** Genome browser snapshot showing RRBS-captured CpG sites (short blue, purple or red lines) of the indicated samples in *ESC-specific loci* (i.e., *Slc15a1* and *Tex19.2*). Scale bar indicates methylation levels ranging from no methylation (blue), intermediate methylation (purple) to maximum methylation (red).

**
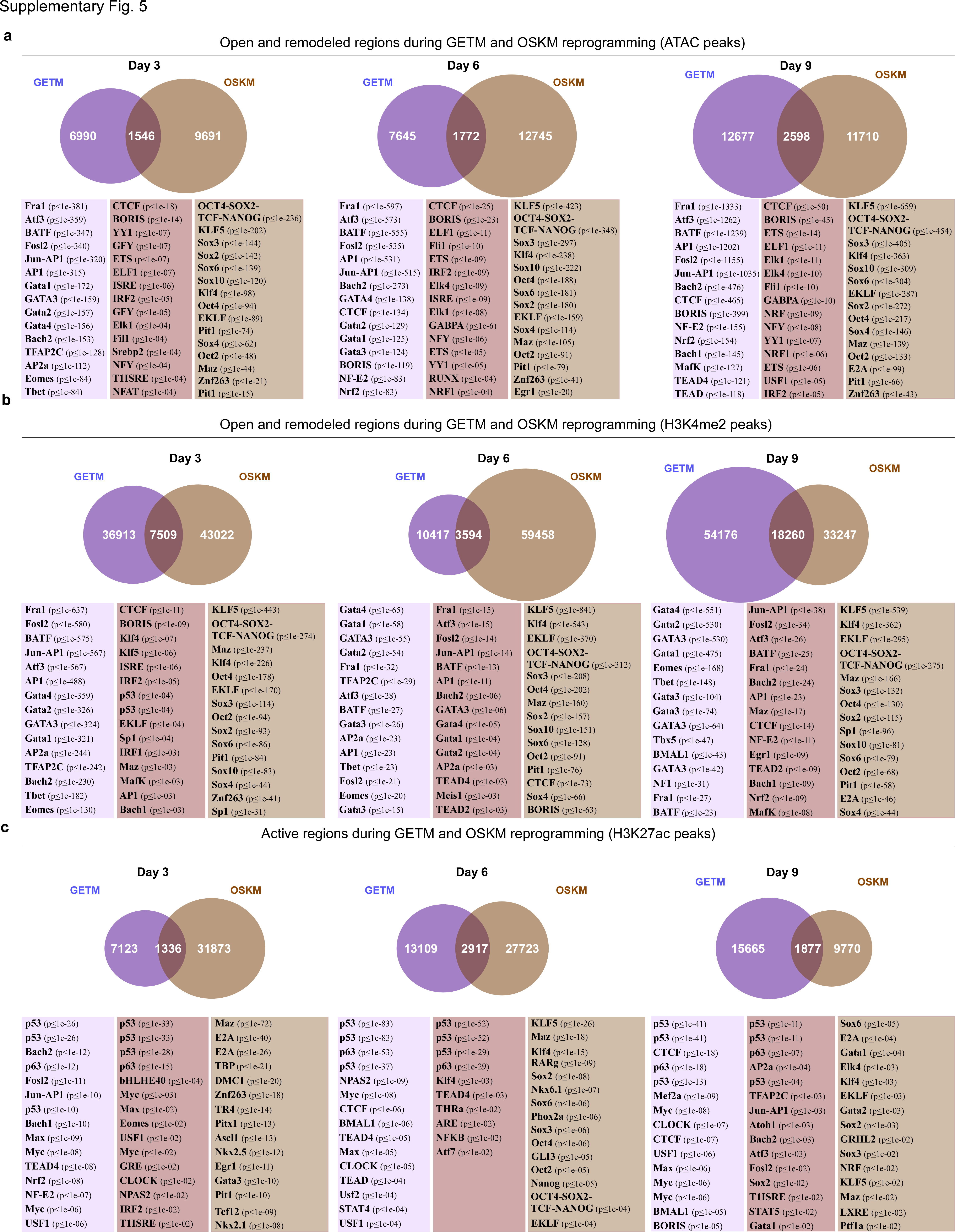
**

**Supplementary Fig. 5. Motif enrichment of ATAC, H3K27ac and H3K4me2 peaks during GETM and OSKM reprogramming. (a-c)** Venn diagrams and motif analysis for ATAC-seq peaks (a), H3K4me2 peaks (b) and H3K27ac peaks (c). Comparison of GETM-only (left wedge, purple), GETM and OSKM (interaction), and OSKM-only (right wedge, brown) peaks from day 3 to day 9. Below are motifs, differentially enriched between each set of peaks versus the rest.

**
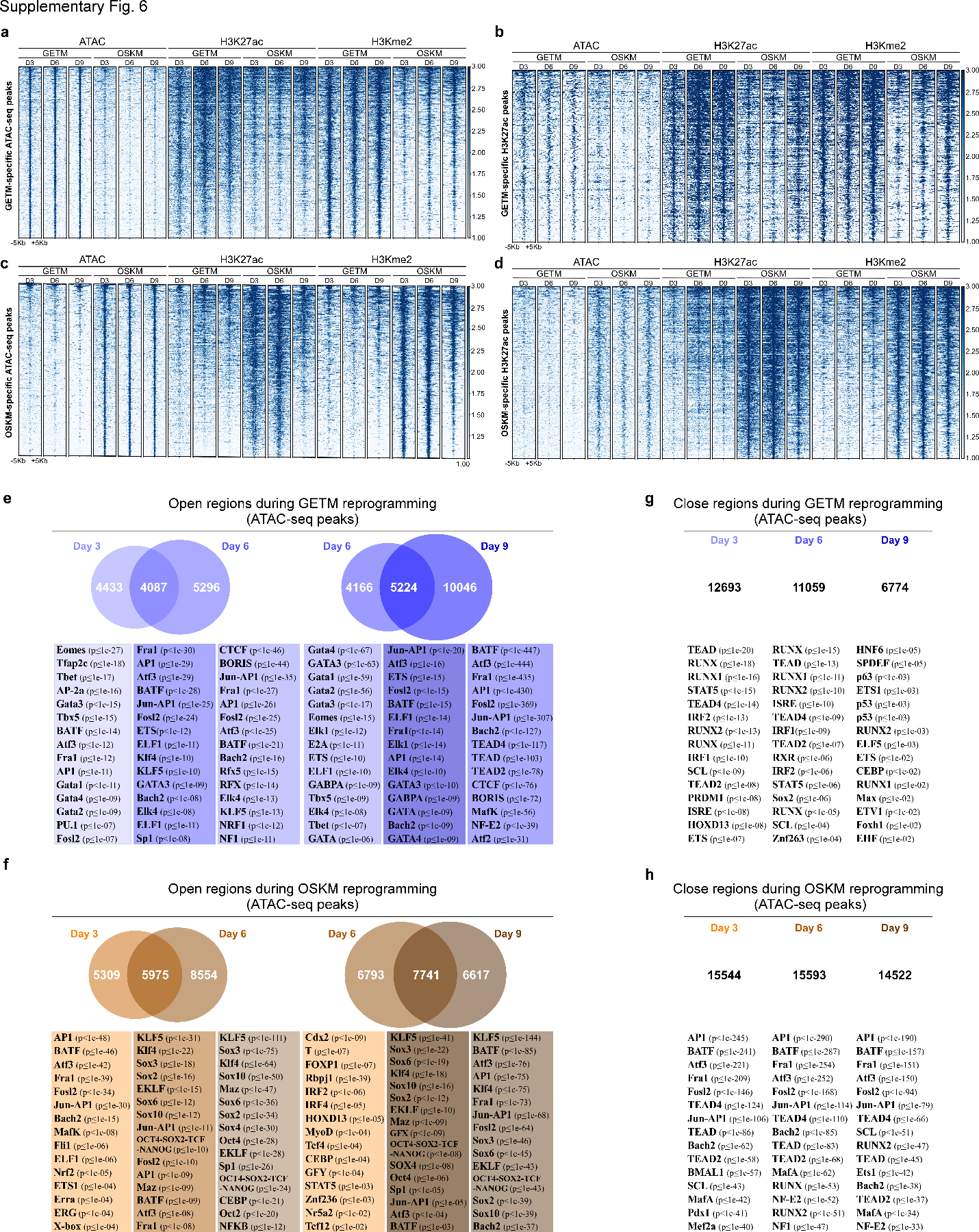
**

**Supplementary Fig. 6. Unique behavior and motif enrichment of ATAC, H3K27ac and H3K4me2 peaks during GETM and OSKM reprogramming. (a-d)** Heatmap showing ATAC-seq and ChIP-seq (H3K27ac and H3K4me2) across 1716 differential ATAC-seq peaks (p<1e-3) in GETM (a and b) or 2848 OSKM (c and d). Shown are genomic regions of peak locations ±5Kb. Differential peaks were called using DESeq2 analysis on the number of reads in each of 18,421 ATAC-seq peaks, using a significance threshold of adjusted p-value < 1e-3. **(e)** Comparison of GETM ATAC-seq peaks from days 3 and 6 (left) or 6 and 9 (right). Shown below are enriched motifs for each binary set of peaks. From left to right: day 3-only (left wedge) vs all day 6 peaks; day 3 and 6 peaks (intersection) vs day 3-only and day 6-only peaks; day 6-only (right wedge) vs all day 3 peaks; day 6-only (left wedge) vs all day 9 peaks; day 6 and 9 peaks (intersection) vs day 6-only and Day 9-only peaks; day 9-only (right wedge) vs all day 6 peaks. **(f)** Same as E but for OSKM **(g)** Similar analysis for 12,693 genomic regions that are accessible in MEFs but close on GETM day 3; or 11,059 regions that are accessible in day 3 but close in day 6; or 6774 regions that are accessible in Day 6 but close in day 9 **(h)** Same as (g) but for OSKM. **
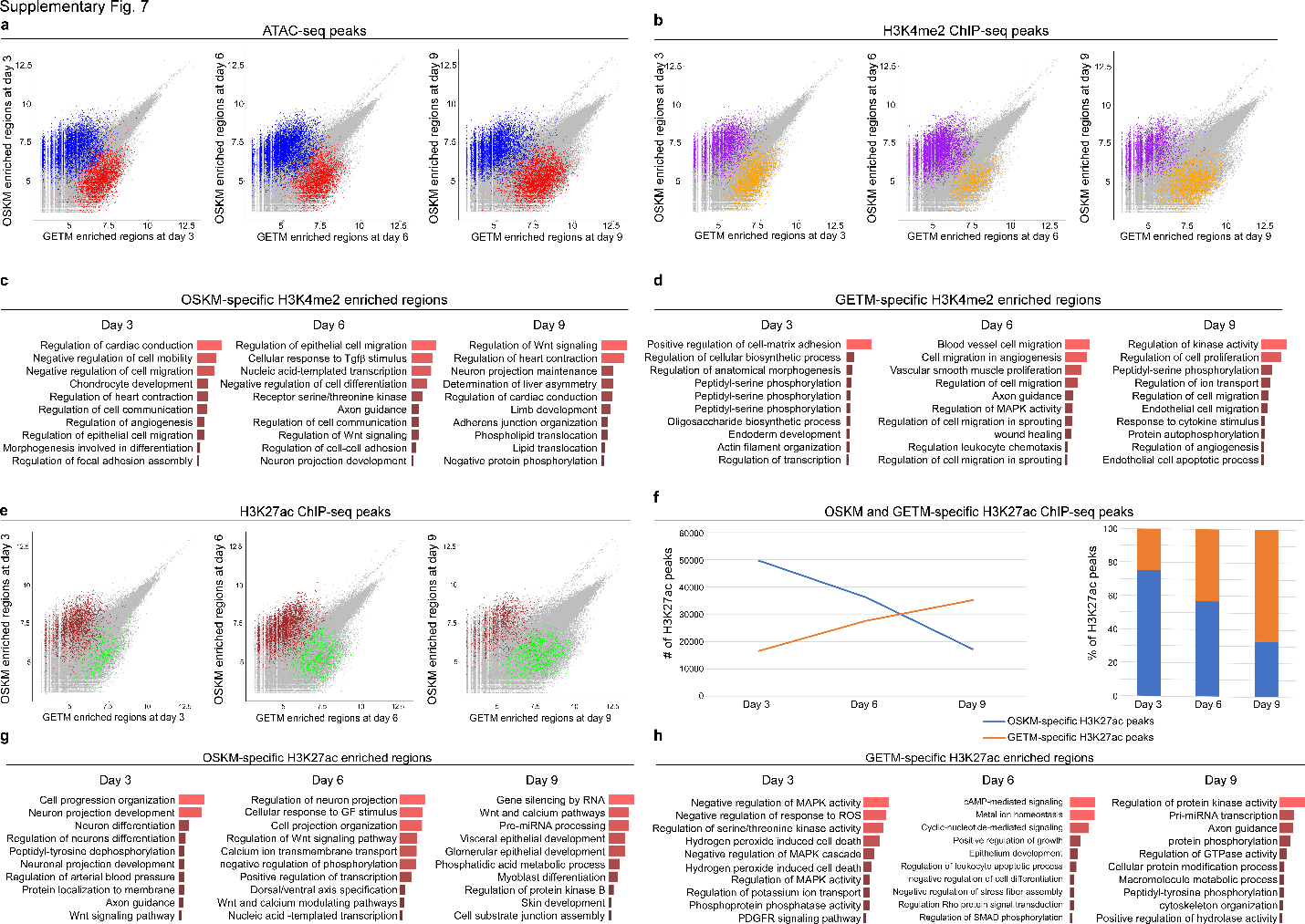
Supplementary Fig. 7. OSKM and GETM enriched regions for ATAC-seq peaks, H3K4me2 and H3K27ac peaks. (a)** Scatter plot of enriched regions differentially accessible between different cellular states during the reprogramming process using GETM and OSKM days 3, 6, and 9. Differential accessible regions are marked for OSKM (blue) and GETM (red) with adjusted p-value < 0.001. **(b)** Scatter plot of enriched regions that are both differentially accessible and enriched for H3K4me2 ChIP-seq differential peaks on top of the ATAC-seq signal during the reprogramming process using GETM and OSKM days 3, 6 and 9. Enriched regions for OSKM (purple) and GETM (orange) are marked. **(c)** Top 10 enriched gene ontology (GO) terms within OSKM-specific regions that are both differentially accessible and enriched for H3K4me2 at days 3, 6 and 9 tested in the biological process ontology. The length of the bar represents the significance of that specific gene-set or term. In addition, the brighter the color, the more significant that term is. **(d)** Top 10 enriched gene GO terms within GETM-specific regions that are both differentially accessible and enriched for H3K4me2 at days 3, 6 and 9 tested in the biological process ontology. **(e)** Scatter plot of enriched regions that are both differentially accessible and enriched for H3K27ac ChIP-seq differential peaks on top of the ATAC-seq signal for enriched regions GETM and OSKM reprogramming at days 3, 6 and 9. Enriched regions are marked with maroon for OSKM and green for GETM. **(f)** Line plot and stacked column chart showing differential dynamics of enrichment for H3K27ac during both OSKM and GETM reprogramming at days 3, 6, 9. **(g)** Top 10 enriched GO terms within OSKM-specific regions that are both differentially accessible and enriched for H3K27ac at days 3, 6 and 9 tested in the biological process ontology. **(h)** Top 10 enriched GO terms within GETM-specific regions that are both differentially accessible and enriched for H3K27ac at days 3, 6 and 9 tested in the biological process ontology. The length of the bar represents the significance of that specific gene-set or term, the brighter the color, the more significant that term is.

**
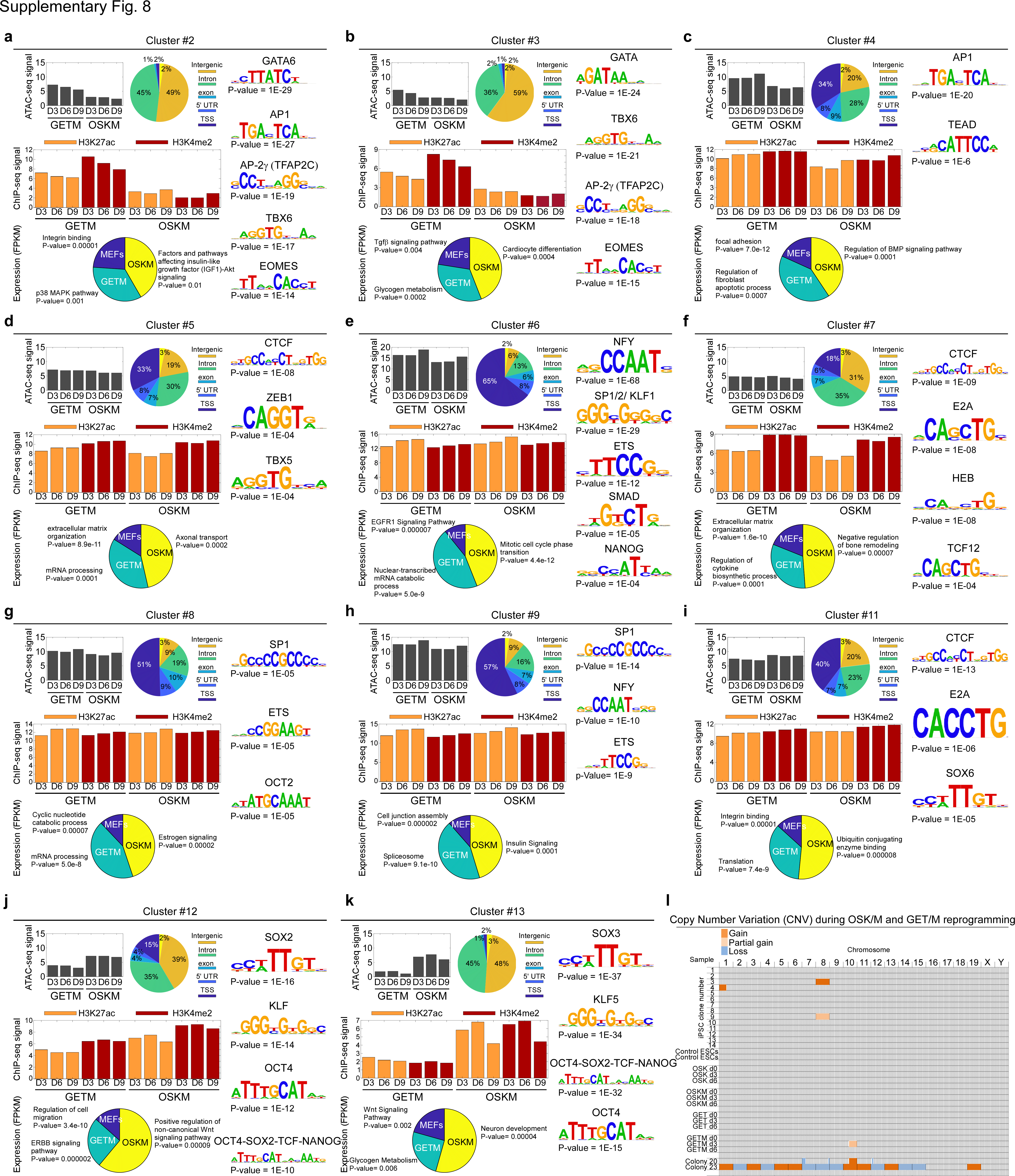
Supplementary Fig. 8. Data integration of chromatin accessibility and activity and gene expression and CNV analysis. (a-k)** 18,420 GETM and OSKM ATAC peaks from days 3, 6, 9 were clustered to 14 clusters. Shown for each cluster are: mean ATAC-seq signal (top left), analysis of their genomic annotations (pie chart, center), enriched transcription factor motifs (right panel), average ChIP-seq signals of H3K27ac and H3K4me2 following GETM and OSKM induction (middle panel), and a pie chart for RNA expression levels and GO terms for genes that are associated with each cluster ATAC-seq peaks and exhibit the highest expression levels in MEFs (blue), or GETM (green) or OSKM (yellow, Bottom panel). **(l)** A graph summarizing the various copy number variations (CNVs) identified in OSK/M or GET/M reprogrammable cells (days 0, 3 and 6) and in isolated iPSC clones. Final TSCs/iTSCs hold an intrinsic capacity to accumulate genomic aberrations^13^ and thus are not measured here. Parental ESC line and partially reprogrammed iPSC colonies number 20 and 23^28^ were used as negative and positive control, respectively. All data were aligned to the parental MEFs.


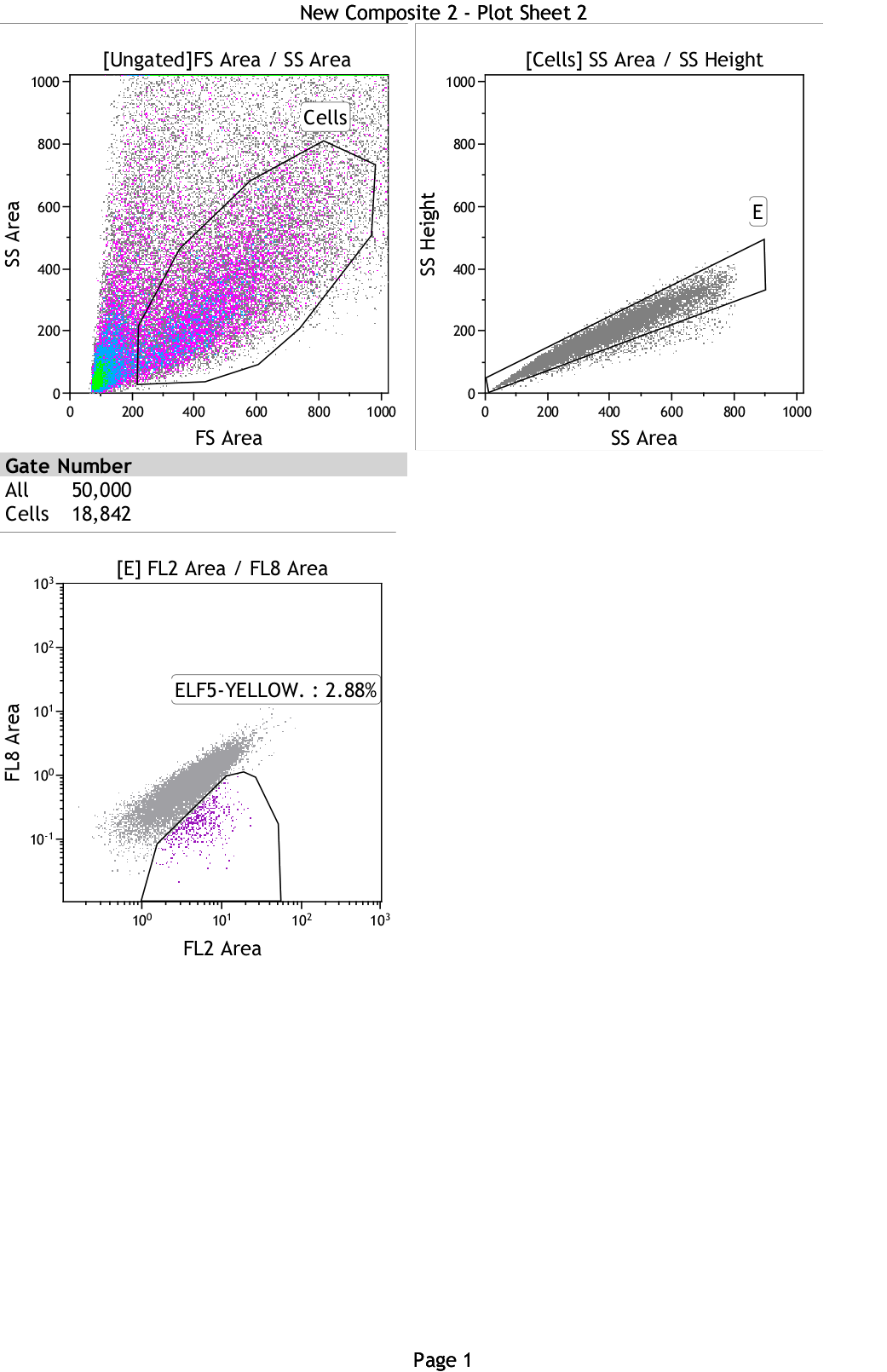


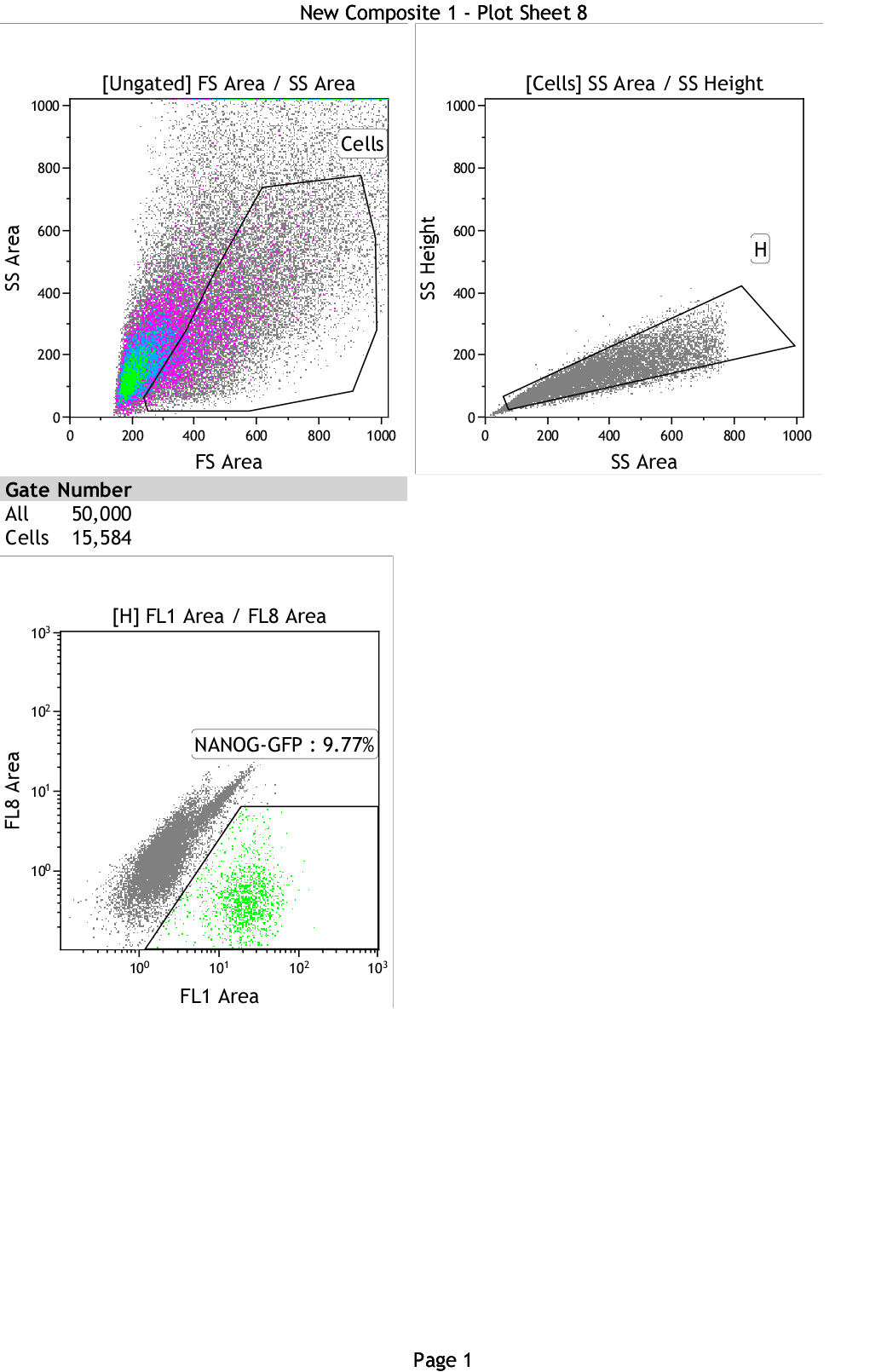


**Supplementary Fig. 9. Gating strategy for iTSC and iPSC reprogramming.** As reprogramming is accompanied by massive cell death, all samples were initially gated using the FSC/SSC gating to identify the live cell population (below 200 FS Area). To remove cell doublets, single cells were selected by gating forward scatter height vs area. The positively fluorescent cells were gated based on the fluorescent intensity as compared to control cells that do not have the fluorescent reporter gene.
