# Supplementary material for: Comparative parallel multi-omics analysis during the induction of pluripotent and trophectoderm states
Source: Nat Commun. 2022 Jun 17;13:3475. doi: 10.1038/s41467-022-31131-8 (PMC9205865; doi:10.1038/s41467-022-31131-8)
Supplement: Supplementary file 2 — Description of Additional Supplementary Files [file 41467_2022_31131_MOESM2_ESM.pdf]

Description of Additional Supplementary Files

Supplementary Data 1. Bulk RNA-seq analysis identifies 27 differentially expressed clusters between GETM and OSKM reprogramming

Gene ontology (GO) analysis of the genes associated with 27 RNA-seq clusters with different transcription expression patterns and interactions.

Supplementary Data 2. Single cell RNA-seq analysis identifies various sub-populations for each reprogramming process

Gene ontology (GO) analysis of the genes associated with the various sub-population identified by EnrichR during GETM and OSKM reprogramming.

Supplementary Data 3. RRBS analysis identifies differentially methylated clusters for each reprogramming process

Gene ontology (GO) analysis of the genes associated with each cluster of DMRs showing unique methylation trends.
