# Supplementary material for: Comparative parallel multi-omics analysis during the induction of pluripotent and trophectoderm states
Source: Nat Commun. 2022 Jun 17;13:3475. doi: 10.1038/s41467-022-31131-8 (PMC9205865; doi:10.1038/s41467-022-31131-8)
Supplement: Supplementary file 6 — Reporting Summary [file 41467_2022_31131_MOESM6_ESM.pdf]

nature research

Corresponding author(s): Yosef Buganim
Tommy Kaplan

Last updated by author(s): 2022/30/05

Reporting Summary

Nature Research wishes to improve the reproducibility of the work that we publish. This form provides structure for consistency and transparency in reporting. For further information on Nature Research policies, see our Editorial Policies and the Editorial Policy Checklist.

Statistics

For all statistical analyses, confirm that the following items are present in the figure legend, table legend, main text, or Methods section.

n/a	Confirmed	
☐	☒	The exact sample size (*n*) for each experimental group/condition, given as a discrete number and unit of measurement
☐	☒	A statement on whether measurements were taken from distinct samples or whether the same sample was measured repeatedly
☐	☒	The statistical test(s) used AND whether they are one- or two-sided<br>*Only common tests should be described solely by name; describe more complex techniques in the Methods section.*
☐	☒	A description of all covariates tested
☒	☐	A description of any assumptions or corrections, such as tests of normality and adjustment for multiple comparisons
☐	☒	A full description of the statistical parameters including central tendency (e.g. means) or other basic estimates (e.g. regression coefficient) AND variation (e.g. standard deviation) or associated estimates of uncertainty (e.g. confidence intervals)
☐	☒	For null hypothesis testing, the test statistic (e.g. *F*, *t*, *r*) with confidence intervals, effect sizes, degrees of freedom and *P* value noted<br>*Give P values as exact values whenever suitable.*
☐	☒	For Bayesian analysis, information on the choice of priors and Markov chain Monte Carlo settings
☐	☒	For hierarchical and complex designs, identification of the appropriate level for tests and full reporting of outcomes
☒	☐	Estimates of effect sizes (e.g. Cohen's *d*, Pearson's *r*), indicating how they were calculated

Our web collection on statistics for biologists contains articles on many of the points above.

Software and code

Policy information about availability of computer code

Data collection	Bulk RNA seq were collected with Illumina NextSeq 500 platform to generate 75-bp single-end reads. Single-cell RNA-seq using the 10x Genomics platform and libraries were sequenced using Illumina NextSeq 500 platform. The RRBS libraries were sequenced by Illumina HiSeq 2000 platform. ChIP and ATAC data were obtained using Illumina NextSeq 500 platform.
Data analysis	Bulk RNA-seq<br>Low quality bases and sequencing adaptors of 36 raw fastq files RNA-seq containing single-end 61bp-long reads were trimmed using Trim Galore (V 0.6.0, https://github.com/FelixKrueger/TrimGalore) and then mapped to the mm9 reference genome using HISAT2 (V 2.1.0, 81) with default parameters. Read counting was performed using featureCounts (V 1.6.2, 82) with (Mus_musculus.NCBI37.gtf annotation). Differential gene expression analysis was performed using DESeq2_1.26.0 package82. Unsupervised hierarchical clustering was performed for 10,000 most variable genes among ESCs, bdTSCs, fibroblasts and cells during reprogramming. R package dynamicTreeCut83 was used to perform adaptive branch pruning detecting 27 prominent clusters. R packages Enrichr (V 2.1, 84) and ClusterProfiler (V 3.14.3, 85) were used to query Biological processes, Mouse gene atlas and KEGG pathways analysis of significantly over-represented genes for each cluster. A second aligner TopHat [4] (V 2.0.6, 86) was used to map reads to mm9 reference genome. Mapped reads were then processed using cufflinks [4] (V 2.0.2, 87), and gene expression levels (FPKM) were calculated for each replicate. Differential gene expression analysis was performed using DESeq2 package (V 1.26).<br><br>Single-cell RNA-seq<br>scRNA-seq libraries were generated from each time point using the 10X Genomics. The cellranger-6.1.1 [https://github.com/10XGenomics/cellranger] was used for mapping of the 10x single-cell RNA-seq data. Read1 data of pooled cells were split into single-cell data using the barcode sequences contained in the first 16 bps. The next 10 bps were recorded as unique molecular identifiers (UMIs). Read2 with 75 bp were aligned to the mm10 reference genome. We used Seurat (V 4.0.5, 88) to pre-processing the data and perform clustering. The function 'FindAllMarkers' to identify the marker genes for each of the clusters in the UMAP representation. For day 3 OSKM and GETM reprogramming and iTSCs, 20,000 cells were profiled. Around 7,000 cells from each reprogramming system were pooled with 3,000 cells of Sox2-GFP stable

iTSCs cultured on feeder cells under TSC defined medium, TX30. Initial clustering for all the dataset was done to explore and determine quality control cutoffs. Clusters that had an average UMIs ≤10,000 or an average mitochondrion UMIs >10% were excluded. For day 6 OSKM and GETM reprogramming, we excluded clusters that had an average UMIs <8,000 or average mitochondrion UMIs >10%. For day 12 OSKM and GETM reprogramming, we excluded clusters that had an average UMIs <9,000 or average mitochondrion UMIs >10%. R package DoubletFinder (V2.0.3), [https://github.com/chris-mcginnis-ucsf/DoubletFinder] was used to identify and exclude potential doublets. Following quality control (i.e. removal of duplets, lowly expressed cells and mitochondrial RNA-enriched cells) we analyzed a sum of 26,839 cells for the 7 conditions: OSKM: (D3 :4952, D6: 3181, D12: 2835 cells), GETM (D3 :4756, D6: 4625, D12: 3097 cells) and iTSCS: (3393 cells).

DNA methylation
Low quality bases and sequencing adaptors of 45 raw fastq files were trimmed using Trim galore (V 0.6.0, https://github.com/FelixKrueger/TrimGalore) and then mapped to the mm9 reference genome using Bsmap (V 2.90, 88) with flags -S 10 -R -p 8 -D C-CGG. Bam files belonging to same reprogramming system and day were merged to ensure maximum overlap between all samples. Methylation beta values were extracted from the BAM files using wgbs_tools (https://github.com/nloyfer/wgbs_tools). Methylation markers were identified using in-house developed script find_markers.py to generate Bed files with p-value < 0.05 between different conditions summarized in different groups. 130,000 blocks were identified with significant methylation alteration that occurs during reprogramming in both OSKM and GETM reprogramming. In order to minimize noise and extract significant trends, we used the K-means algorithm to classify ~130,000 blocks that are shared amongst all samples during reprogramming to a TSC or pluripotent states and obtained 100 clusters. A new table was constructed by averaging DNA methylation levels per sample per cluster and then projected the processed data onto the first two principal components. Clusters loading plot showed significant clusters contributed to the first two principal components and clusters that are near to each other showed similar trends of methylation allowing us to extract 15 different trends shown as heatmaps in Fig. 4a and Fig. S4a. Genomic regions associated with all blocks belonging to each of the 15 clusters were annotated using GREAT (V 4.0.432) and were summarized in Supplementary data file 3.

ATAC and ChIP
Data were mapped to the mm9 reference using bwa (V 0.7.17-r1188). The mapped reads were converted to BAM format and filtered by mapping quality (MAPQ) of >=10, retaining only properly aligned pairs (samtools -F 1796 flag). The BAM files were then sorted and indexed using samtools (V 1.9). Bigwig coverage tracks were generated using deepTools bamCoverage (V 3.4.1) with the following flags: --normalizeUsing RPGC -bs 50 -e 500 --effectiveGenomeSize 2150570000. Coverage peaks were called using MACS (V 2.1.2) with flags -g mm --slocal=2000 --llocal=20000 --nomodel --extsize=300 -f BAMPE. Peaks of multiple replicates were considered if the peak was shared by at least 30% of the replicates. Peaks from each experiment were then divided into subsets, including peaks that appear in both OSKM and GETM (3, 6, or 9 days after induction) but not in MEFs, peaks from GETM (days 3,6,9) not identifiable in MEFs, OSKM peaks (days 3, 6, 9) not identifiable in MEFs, and disjoint sets of cell-type specific peaks (e.g. GETM day 3 peaks not found in MEFs or in OSKM day 3, etc.). We also analyzed peaks from ESC, TSC or MEF cells. Genomic regions from each group of peaks were then annotated using annotatePeaks.pl (HOMER suite, http://homer.ucsd.edu/homer/ngs/annotation.html, UCSC mm9 genome version) as Promoter, TTSs, 5' and 3' UTRs, or as Exonic, Intronic, or Intergenic regions. Motif analysis was done using HOMER function "findMotifsGenome.pl -nomotif" for peaks overlapping from replicates separately. Peaks overlapping MEF peaks (top 50K) were then removed. Finally, the center 250bp of each peak was considered for further analysis (peaks shorter than 250bp were removed). We further divided the peaks of each time point into disjoint groups, including peaks identified in both GETM and OSKM ATAC-seq (e.g. GETM&OSKM D03), GETM-only peaks (e.g. GETM\OSKM D03) or OSKM-only peaks (e.g. OSKM\GETM D03). A similar approach was applied to H3K27ac and H3K4me2 ChIP-seq peaks.

Flow cytometry
Flow cytometry experiments were analyzed by Beckman Coulter flow cytometer using the Kaluza Software (V 1.0.14029.14028).

For manuscripts utilizing custom algorithms or software that are central to the research but not yet described in published literature, software must be made available to editors and reviewers. We strongly encourage code deposition in a community repository (e.g. GitHub). See the Nature Research guidelines for submitting code & software for further information.

Data

Policy information about availability of data

All manuscripts must include a data availability statement. This statement should provide the following information, where applicable:

- Accession codes, unique identifiers, or web links for publicly available datasets
- A list of figures that have associated raw data
- A description of any restrictions on data availability

Data availability
ATAC-seq, ChIP-seq, RRBS, scRNA-seq, bulk RNA-seq and CNV has been deposited to the Gene Expression Omnibus database (GEO, Accession number GSE171127 [GSE171127 [https://www.ncbi.nlm.nih.gov/geo/query/acc.cgi?acc=GSE171127]). Additional data from Benchetrit et al. are available at GEO, GSE98124 [https://www.ncbi.nlm.nih.gov/geo/query/acc.cgi?acc=GSE98124]. All analyses used UCSC mm9 mouse reference genome [http://genome.ucsc.edu/cgi-bin/hgGateway?db=mm9], except for the 10x single-cell RNA-seq data, which used mm10 [http://genome.ucsc.edu/cgi-bin/hgGateway?db=mm10]. The figures that are associated with the raw data files are: Figs 2a-m, 3a-i, 4a-e, 5a-j, 6a-b, 7a-i and supplementary Figs: 1e-j, 2a-g, 3a-b, 3e-f, 4a-g, 5a-c, 6a-h, 7a-h, 8a-i. Remaining data are provided within the Article, Supplementary Information and Source Data.

Field-specific reporting

Please select the one below that is the best fit for your research. If you are not sure, read the appropriate sections before making your selection.

- [x] Life sciences
- [ ] Behavioural & social sciences
- [ ] Ecological, evolutionary & environmental sciences

For a reference copy of the document with all sections, see nature.com/documents/nr-reporting-summary-flat.pdf

Life sciences study design

All studies must disclose on these points even when the disclosure is negative.

Sample size	The sample size was chosen on the basis of prior studies that showed significant results with similar sample sizes.<br>1- Tran, Khoa A et al. "Defining Reprogramming Checkpoints from Single-Cell Analyses of Induced Pluripotency." Cell reports vol. 27,6 (2019): 1726-1741.e5. doi:10.1016/j.celrep.2019.04.056.<br>2-Knaupp, Anja S et al. "Transient and Permanent Reconfiguration of Chromatin and Transcription Factor Occupancy Drive Reprogramming." Cell stem cell vol. 21,6 (2017): 834-845.e6. doi:10.1016/j.stem.2017.11.007.
Data exclusions	No data were excluded from the analysis.
Replication	Replicate experiments were successful.<br>Two independent biological replicates were analyzed for each timepoint/sample for each high throughput assay (bulk RNA-seq, RRBS, ATAC-seq and ChIP-seq) using 5 independent reprogramming experiments. For sc-RNA-seq, 3 independent reprogramming experiments were conduced in triplicates. for each timepoint/sample the three replicates were mixed and run through the 10x genomics. The three experiments were run in 4 independent lanes while GETM samples are mixed with OSKM samples to avoid batch effect.<br>For all the other analyses, two or more biological replicates were used in three or more independent repetitions.
Randomization	Randomization was not needed since all the experiments were done in cell culture. No animals were used in this study.
Blinding	Blinding was applied for counting the number of stable iTSC/iPSC colonies from the different conditions in the same experiment (Figs. 2o and 6e-g).

Reporting for specific materials, systems and methods

We require information from authors about some types of materials, experimental systems and methods used in many studies. Here, indicate whether each material, system or method listed is relevant to your study. If you are not sure if a list item applies to your research, read the appropriate section before selecting a response.

Materials & experimental systems

n/a	Involved in the study	
☐	☒	Antibodies
☐	☒	Eukaryotic cell lines
☒	☐	Palaeontology and archaeology
☒	☐	Animals and other organisms
☒	☐	Human research participants
☒	☐	Clinical data
☒	☐	Dual use research of concern

Methods

n/a	Involved in the study	
☐	☒	ChIP-seq
☐	☒	Flow cytometry
☒	☐	MRI-based neuroimaging

Antibodies

Antibodies used	For Immunofluorescence: anti-CDX2 (Biogenex, CDX2-88, 1:1000) and anti-TACSTD2 (TROP2, Abcam, Ab214488, 1:500). Secondary antibodies: Goat Anti-Mouse IgG (Alexa Fluor 488, Ab150113, 1:500) and Goat Anti-Rabbit IgG (Alexa Fluor 594, Ab150080, 1:500).<br>For ChIP-seq: anti-H3K27ac antibody (Abcam, ab4729, 2microgram/reaction) and anti-H3K4me2 antibody (Millipore, 07-030, 2microgram/reaction).
Validation	The Immunofluorescence antibodies were validated on control mouse embryonic fibroblasts or TSCs prior to use for experiment.<br>Anti-CDX2 (Biogenex, CDX2-88, 1:000) antibody were used before by our lab: Benchetrit, Hana et al. "Direct Induction of the Three Pre-implantation Blastocyst Cell Types from Fibroblasts." Cell stem cell vol. 24,6 (2019): 983-994.e7. doi:10.1016/j.stem.2019.03.018.<br>anti-TACSTD2(TROP2, Abcam, Ab214488, 1:500) antibody were used by other groups and validated in our lab: Yao, Catherine D et al. "AP-1 and TGFß cooperativity drives non-canonical Hedgehog signaling in resistant basal cell carcinoma." Nature communications vol. 11,1 5079. 8 Oct. 2020, doi:10.1038/s41467-020-18762-5.<br>anti-H3K27ac antibody (Abcam, ab4729, 2microgram/reaction) was validated for ChIP-seq by more than 1000 citation:<br>Williams K et al. Skeletal muscle enhancer interactions identify genes controlling whole-body metabolism. Nat Commun 11:2695 (2020).<br>anti-H3K4me2 antibody (Millipore, 07-030, 2microgram/reaction) was validated for ChIP-seq by more than 100 citation:<br>Stadtfeld M et al. Aberrant silencing of imprinted genes on chromosome 12qF1 in mouse induced pluripotent stem cells. Nature 465 175-81 2010.

Eukaryotic cell lines

Policy information about cell lines

Cell line source(s)	BYKE mouse embryonic fibroblasts were derived in our laboratory, more info about the cells: Benchetrit H et al. Direct Induction of the Three Pre-implantation Blastocyst Cell Types from Fibroblasts. Cell Stem Cell. 2019 Jun 6;24(6):983-994.e7. doi: 10.1016/j.stem.2019.03.018. Epub 2019 Apr 25. PMID: 31031139; PMCID: PMC6561721.
Authentication	The BYKE mouse embryonic fibroblasts were not authenticated
Mycoplasma contamination	All cell lines were tested for mycoplasma contamination.
Commonly misidentified lines (See ICLAC register)	BYKE mouse embryonic fibroblasts, The cells were generated in our laboratory, they contain a unique fluorescent reporter genes that can distinguish between the Pluripotent and TSC States.

ChIP-seq

Data deposition

- [x] Confirm that both raw and final processed data have been deposited in a public database such as GEO.
- [x] Confirm that you have deposited or provided access to graph files (e.g. BED files) for the called peaks.

Data access links *May remain private before publication.*	All data are available through GEO accession number: GSE171127 https://www.ncbi.nlm.nih.gov/geo/query/acc.cgi?acc=GSE171127
Files in database submission	All Fastq files for called peaks are available.
Genome browser session (e.g. UCSC)	http://genome-euro.ucsc.edu/s/tomkap/mm9_Buganim2

Methodology

Replicates	Two biological replicates were sequenced for each sample in any given experiment.
Sequencing depth	The Sequencing was paired-end, total of 75 bp reads from both sides. The depth for each experiment was between 20 and 40 million reads per replicate.
Antibodies	The antibodies that were used are mentioned at the methods section.<br>anti-H3K27ac antibody (Abcam, ab4729, 2microgram/reaction) was validated for ChIP-seq by more than 1000 citation:<br>Williams K et al. Skeletal muscle enhancer interactions identify genes controlling whole-body metabolism. Nat Commun 11:2695 (2020).<br>anti-H3K4me2 antibody (Millipore, 07-030, 2microgram/reaction) was validated for ChIP-seq by more than 100 citation:<br>Stadtfeld M et al. Aberrant silencing of imprinted genes on chromosome 12qF1 in mouse induced pluripotent stem cells. Nature 465 175-81 2010.
Peak calling parameters	Bigwig coverage tracks were generated using deepTools bamCoverage (v 3.4.1) flags: --normalizeUsing RPGC -bs 50 -e 500 --effectiveGenomeSize 2150570000. Coverage peaks were called using MACS (V 2.1.2) with flags -g mm --slocal=2000 --llocal=20000 --nomodel --extsize=300 -f BAMPE.
Data quality	Peaks were called using MACS for each ChIP and ATAC-seq experiment separately (see details below, with default FDR threshold of 0.05), yielding 50K-120K peaks. To ensure data quality and reproducibility, we then compared the called peaks across replicates, and retained only peaks that were identified (overlapped) by at lease 30% of replicates.
Software	For the initial peak calling, we used MACS (v2.1.2 103) with flags -g mm --slocal=2000 --llocal=20000 --nomodel --extsize=300 -f BAMPE

Flow Cytometry

Plots

Confirm that:

- [x] The axis labels state the marker and fluorochrome used (e.g. CD4-FITC).
- [x] The axis scales are clearly visible. Include numbers along axes only for bottom left plot of group (a 'group' is an analysis of identical markers).
- [x] All plots are contour plots with outliers or pseudocolor plots.
- [x] A numerical value for number of cells or percentage (with statistics) is provided.

Methodology

Sample preparation	Cells were trypsinized, washed with PBSx1 and filtered through mesh paper. Samples were analyzed by a Beckman Coulter (Gallios) flow cytometer using the Kaluza Software.
Instrument	Beckman Coulter FACS
Software	Kaluza Software (V 1.0.14029.14028).
Cell population abundance	Cell sorting not employed.
Gating strategy	All samples were initially gated using the FSC/SSC gating to identify the live cell population, and then single cells were selected by gating forward scatter height vs area. The positively fluorescent cells were gated based on the fluorescent intensity as compared to control cells that do not have the fluorescent reporter gene.

☒ Tick this box to confirm that a figure exemplifying the gating strategy is provided in the Supplementary Information.
